# Association Analysis of Variants of *DSCAM* and *BACE2* With Hirschsprung Disease Susceptibility in Han Chinese and Functional Evaluation in Zebrafish

**DOI:** 10.3389/fcell.2021.641152

**Published:** 2021-05-31

**Authors:** Yan-Jiao Lu, Wen-Wen Yu, Meng-Meng Cui, Xian-Xian Yu, Huan-Lei Song, Mei-Rong Bai, Wen-Jie Wu, Bei-Lin Gu, Jun Wang, Wei Cai, Xun Chu

**Affiliations:** ^1^Department of Pediatric Surgery, Xinhua Hospital Affiliated to Shanghai Jiao Tong University School of Medicine, Shanghai, China; ^2^Shanghai Key Laboratory of Pediatric Gastroenterology and Nutrition, Shanghai, China; ^3^Shanghai Institute of Pediatric Research, Shanghai, China

**Keywords:** Hirschsprung disease, *DSCAM*, *BACE2*, zebrafish model, enteric nervous system

## Abstract

Hirschsprung disease (HSCR) has a higher incidence in children with Down syndrome (DS), which makes trisomy 21 a predisposing factor to HSCR. *DSCAM* and *BACE2* are close together on the HSCR-associated critical region of chromosome 21. Common variants of *DSCAM* and rare variants of *BACE2* were implicated to be associated with sporadic HSCR. However, the submucosal neuron defect of DS mouse model could not be rescued by normalization of *Dscam*. We aimed to explore the contribution of *DSCAM* and *BACE2* to the development of the enteric nervous system (ENS) and HSCR susceptibility. We genotyped 133 tag single-nucleotide polymorphisms (SNPs) in *DSCAM* and *BACE2* gene region in 420 HSCR patients and 1,665 controls of Han Chinese. Expression of *DSCAM* and *BACE2* homologs was investigated in the developing gut of zebrafish. Overexpression and knockdown of the homologs were performed in zebrafish to investigate their roles in the development of ENS. Two *DSCAM* SNPs, rs430255 (*P*_*Addtive*_ = 0.0052, *OR* = 1.36, 95% CI: 1.10–1.68) and rs2837756 (*P*_*Addtive*_ = 0.0091, *OR* = 1.23, 95% CI: 1.05–1.43), showed suggestive association with HSCR risk. Common variants in *BACE2* were not associated with HSCR risk. We observed *dscama*, *dscamb*, and *bace2* expression in the developing gut of zebrafish. Knockdown of *dscama*, *dscamb*, and *bace2* caused a reduction of enteric neurons in the hindgut of zebrafish. Overexpression of *DSCAM* and *bace2* had no effects on neuron number in the hindgut of zebrafish. Our results suggested that common variation of *DSCAM* contributed to HSCR risk in Han Chinese. The dysfunction of both *dscams* and *bace2* caused defects in enteric neuron, indicating that *DSCAM* and *BACE2* might play functional roles in the occurrence of HSCR. These novel findings might shed new light on the pathogenesis of HSCR.

## Introduction

Hirschsprung disease (HSCR) is a highly heritable disorder, which mainly results from the failure of neural crest cells to fully colonize the gut during embryonic development. The incidence of HSCR is estimated at about 1 out of 5,000 live births worldwide (Amiel et al., 2008; Gui et al., 2017; Tilghman et al., 2019). The sporadic form is the majority and accounts for about 80% of all HSCR cases. Up to 20% of HSCR cases are familial with complex inheritance patterns. In 30% of HSCR cases, patients have coexisting congenital syndrome, the most frequent of which is Down syndrome (DS) (Heanue and Pachnis, 2007). HSCR is a highly heritable disorder caused by multiple factors. Previous genetic studies have identified more than 500 mutations in dozens of genes, and common variants in several genes account for about 25% of the overall genetic risk (Emison et al., 2005; Amiel et al., 2008; Garcia-Barcelo et al., 2009; Jiang et al., 2015; Gui et al., 2017; Tang et al., 2018; Tilghman et al., 2019). These disease genes mainly belong to the receptor tyrosine kinase (*RET*) activation pathway, the endothelin receptor, type b (*EDNRB*) signaling pathway, and transcription factors during enteric nervous system (ENS) formation. *RET* is the most important gene, carrying > 80% of all known risk variants.

It is worth noting that HSCR has an incidence of about 1/40 in children with DS, and the risk of HSCR in the DS population is 50 to 100 times that of the general population (Heuckeroth, 2015; Schill et al., 2019). DS is caused by the trisomy of human chromosome 21, which increases the risk of HSCR, suggesting that one or more genes on chromosome 21 contribute to HSCR etiology (Amiel et al., 2008). In 2009, Korbel and colleagues revealed that a discrete critical region <13 Mb might be involved in DS HSCR by using a state-of-the-art genomics method in DS patients carrying rare segmental trisomies of various regions of human chromosome 21 (Korbel et al., 2009). This study suggested that at least one gene in the interval increased the incidence of HSCR. The HSCR-associated critical region contains about 160 coding genes including DS cell adhesion molecule (*DSCAM*) and β-secretase 2 (*BACE2*) (Korbel et al., 2009).

In 2013, an association analysis with 10,895 single-nucleotide polymorphisms (SNPs) in 26 Caucasian DS HSCR cases and their parents identified two associated SNPs (rs2837770 and rs8134673) in intron 3 of *DSCAM*, and the results were replicated in 220 Caucasian cases with isolated HSCR and their parents (Jannot et al., 2013). Subsequently, the association of *DSCAM* with isolated HSCR was confirmed in a South Chinese sample set (Wang et al., 2018). These findings suggested *DSCAM* as a risk gene for HSCR. However, to explore how trisomy 21 affects ENS development, a recent study evaluated the ENS in two DS mouse models, namely, Ts65Dn and Tc1, which are trisomic for many chromosomes 21 homologous genes, including *Dscam* and *Bace2*. Both Ts65Dn and Tc1 mice have markedly reduced submucosal plexus neuron number; however, normalizing the copy number of *Dscam* does not rescue the defect. Therefore, the hypothesis of *DSCAM* underlying the risk of trisomy 21 for HSCR was challenged (Schill et al., 2019).

A recent whole-genome sequence analysis in 464 patients with sporadic S-HSCR and 498 controls revealed that a significant excess of rare protein-altering variants in *BACE2* were associated with HSCR. Knockdown or inhibition of *BACE2* rescued migration defects in human embryonic stem cell-derived ENS precursors. Further functional assays suggested that variants in *BACE2* had a role in protecting enteric neurons from apoptosis (Tang et al., 2018).

Due to the complexity of *DSCAM* and *BACE2* associations with HSCR risk, it is necessary to investigate the associations in independent samples with high resolution. Thereby, we performed an association analysis with tag SNPs covering *DSCAM* and *BACE2* gene region in a Chinese sample set including sporadic HSCR patients and healthy controls. Zebrafish has emerged as a powerful model to assess the function of candidate HSCR genes on ENS development (Kuil et al., 2020). We further used zebrafish model to test the role of *DSCAM* and *BACE2* in ENS development. Homology searches of the zebrafish genome show that zebrafish have two human *DSCAM* homologs (*dscama* and *dscamb*) and one human *BACE2* homolog (*bace2*). We used overexpression, morpholino-based knockdown, and rescue analysis in zebrafish model to investigate the functions of *dscams* and *bace2* in ENS development.

## Materials and Methods

### Study Populations

A total of 420 unrelated sporadic HSCR patients were studied, which included 322 males and 98 females with the male:female ratio of 3.29:1. Diagnosis of HSCR was based on histopathological criteria for HSCR: (1) absence of enteric plexuses with histological evaluation of the aganglionic tract and (2) increased acetylcholinesterase immunohistochemical staining in the nerve fibers. All patients were sporadic cases and had HSCR as the sole phenotype. Patients were classified into three subgroups based on the segment length of aganglionosis: 323 S-HSCR, 58 L-HSCR, and 39 total colonic aganglionosis (TCA). We randomly selected 1,665 gender- and ethnicity-matched healthy individuals who visited Xinhua Hospital for routine health checkup, as controls including 1,281 males and 384 females (the male:female ratio of 3.34:1). HSCR patients and unrelated controls were all Han Chinese and recruited in Xinhua Hospital Affiliated to Shanghai Jiao Tong University School of Medicine. The study was performed according to principles of the Declaration of Helsinki, and the study protocol was approved by the institutional review board of Xinhua Hospital (IRB: XHEC-WSJSW-2018-029). All participants or their parents signed an informed consent form. Genomic DNA was extracted from peripheral blood leukocytes using the QIAamp DNA Blood Mini Kit according to the manufacturer’s protocol (Qiagen, Hilden, Germany).

### Single-Nucleotide Polymorphism Selection and Genotyping

Two *DSCAM* SNP (rs2837770 and rs8134673) implicated to be associated with HSCR in previous studies were selected for replication. Tag SNPs were selected using The Genome Variation Server^1^ based on the HapMap CHB (Han Chinese in Beijing) data. We selected 112 tag SNPs including rs2837770 and rs8134673 with the criteria of minor allele frequency (MAF) ≥ 0.01 and *r*^2^ ≥ 0.8 to cover *DSCAM* region. A total of 21 *SNPs* were selected to cover *BACE2* gene region with the criteria of MAF ≥ 0.01 and *r*^2^ ≥ 0.8. We genotyped the 133 tag SNPs to investigate the associations of *DSCAM* and *BACE2* with HSCR susceptibility. SNPs were genotyped using a Fluidigm platform (Fluidigm Corp., CA). Allele-specific fluorescent (FAM or VIC) primers and common reverse primers were employed for genotyping, and EP1 SNP Genotyping Analysis software was used to analyze the data. We placed one duplicate sample to each 96-well sample plate to assess genotyping accuracy.

Association analysis was performed using PLINK 1.09 with additive model (Purcell et al., 2007). The genotype distribution of each SNP was tested for Hardy–Weinberg equilibrium (HWE) in both case and control population. Linkage disequilibrium (LD) structure was examined by Haploview 4.2 program. The functional consequences of the associated SNP were investigated by checking HaploRegv4.1 database (Ward and Kellis, 2011). The study-level significance was *P* < 0.00038 (0.05/133).

### Zebrafish Lines

All zebrafish experiments were performed on AB zebrafish in accordance with protocols approved by the Animal care and Use Committee of Xinhua Hospital. To prevent pigment formation, embryos were treated with 0.003% 1-phenyl-2-thiourea (PTU) (Sigma-Aldrich) at 24 h post-fertilization (hpf) (Qiu et al., 2020).

### RNA Isolation and Quantitative Real-Time Polymerase Chain Reaction

Total RNA was isolated from 15 wild-type zebrafish embryos of different development stages using TRIzol reagent (TaKaRa, Japan). cDNA was synthesized by using the RevertAid Fist Strand cDNA Synthesis kit (Thermo Fisher Scientific, United States). qRT-PCR was performed using SYBR Green (TaKaRa, Otsu, Japan) on a QuantStudio Dx Real-Time PCR Instrument with QuantStudio Dx Software (Applied Biosystems, Foster City, CA, United States). The 18s ribosomal RNA (*18-s*) gene was chosen as the reference gene (McCurley and Callard, 2008). The relative expression levels of each sample were calculated using the RQ formula (*RQ* = 2^–Δ^ ^Δ^ ^*Cq*^) (Bustin et al., 2009); and these assays were carried out in three independent triplicates, with final calculations based on the means of triplicate wells. The sequences of primers are shown in Supplementary Table 1.

### Whole-Mount *in situ* Hybridization for Zebrafish

We constructed the antisense RNA probe against *dscama*, *dscamb*, and *bace2.* Total RNA was extracted from zebrafish embryos at 48 hpf, and cDNA was obtained by RT-PCR. Target fragments of *dscama*, *dscamb*, and *bace2* were amplified using cDNA as template. The primers are shown in Supplementary Table 2. PCR products were run and isolated on 1.2% agarose gel and purified using a QIAquick Gel Extraction Kit (QIAGEN, Germany). The purified products were cloned into pGEM-T Easy Vector (Promega, United States) and used to synthesize antisense RNA probes labeled by DIG RNA labeling mix (Roche, Penzberg, Germany) (Galicia et al., 2018).

Whole-mount *in situ* hybridization (WISH) was performed as previously described (Cunningham and Monk, 2018). Zebrafish embryos were fixed with 4% paraformaldehyde (PFA) in phosphate-buffered saline (PBS) overnight at 4°C, then PFA was removed, and 100% methanol (MeOH) was used to store embryos at −20°C for at least 2 h. Embryos were digested with proteinase K for an appropriate time to allow a better tissue penetration. The embryos were prehybridized for 2–4 h in prehybridized solution without probe at 60°C and then hybridized with Hyb (+) containing 50–200 ng of antisense RNA probes overnight at 60°C. After the probes were removed and strictly washed, embryos were blocked for 1–3 h at room temperature and incubated with Anti-Digoxigenin-AP Fab fragments at 4°C overnight. BM purple AP substrate as developing solution was used to detect the hybridization signals by a microscope (SMZ25, Nikon, Chiyoda, Japan).

### *In vitro* Synthesis of mRNA

The pCS2DEST vector containing the human full-length open reading frame sequences of *DSCAM* was purchased from Addgene (Edie et al., 2018). The full length of zebrafish *bace2* was amplified from total RNA, which was obtained from zebrafish embryos at 48 hpf, and the segment was cloned into pGEM-T Easy Vector and verified by sequencing. The primer sequences for *bace2* amplification are as follows: the forward primer 5′-ATGCGGCTCTACGGGCTACTGCTACT-3′ and the reverse primer 5′-TCATGGGACAATCCTGACCTGTGGGA-3′. After linearization of plasmids, the mMessage mMachine kit (Ambion, TX, United States) was used for transcription at 37°C for 2 h, and lithium chloride was used for further precipitation and purification of mRNA.

### Micro-Injection of mRNA and Morpholino

Embryonic microinjection was performed into one- to four-cell embryos via a gas-driven apparatus; then the embryos were incubated in 28.5°C up to 5 dpf; and during this period, egg water was changed twice a day, and dead eggs and shed shells were removed. In overexpression experiments, 100 pg of human *DSCAM* mRNA and 100 pg of *bace2* mRNA were injected. Splice-blocking morpholino oligonucleotides (SBMOs) were designed to knockdown the expression of *dscama*, *dscamb*, and *bace2* (Supplementary Table 3). The target MO and a standard control MO were purchased from Gene Tools, LLC (OR, United States). MOs were diluted to working concentrations (0.125, 0.25, and 0.375 mM) in sterile double-distilled water, and approximately 2 nl/embryo MO was injected into the blastomeres. In morpholino rescue experiments, 100 pg of *DSCAM* mRNA/embryo and 4 ng of *dscama*-MO/embryo were co-injected, 50 pg of *DSCAM* mRNA/embryo and 2 ng of *dscamb*-MO/embryo were co-injected, and 100 pg of *bace2* mRNA/embryo and 4 ng of *bace2*-MO/embryo were co-injected.

To verify the effectiveness of the splice blocking MOs, gel electrophoresis of PCR products and qRT-PCR were carried out. Total RNA was extracted from SBMO-injected and control MO-injected embryos at 48 hpf (*n* = 15) using RNeasy Mini Kit (Qiagen, Hilden, Germany). cDNA was synthesized using RevertAid Fist Strand cDNA Synthesis kit (Thermo Fisher Scientific, United States). We amplified cDNA sequences across the MO target site by PCR, and the PCR products were visualized by gel electrophoresis (Supplementary Table 4). For qRT-PCR analysis, total RNA was isolated from the 48-hpf embryos (*n* = 40) injected with the control MO, the splicing MOs, and mRNA synthesized *in vitro*. The qRT-PCR analysis was performed on a QuantStudio Dx Real-Time PCR Instrument with QuantStudio Dx Software (Applied Biosystems), and the expression of the target genes was normalized to the housekeeping gene *18s* (Supplementary Tables 5,6). The relative expression of the target genes in the SBMO-injected or mRNA-injected embryos to control MO-injected embryos was determined using methods reported previously (Bustin et al., 2009).

### Whole-Mount Immunohistochemistry

Embryos were raised to 5 dpf at 28.5°C and then fixed overnight by 4% PFA. After a series of washing, decolorization, permeability, and fixation, embryos were blocked with blocking solution for 3 h at 4°C. The HuC/D antibody (Thermo Fisher Scientific, United States) was stained for ENS neurons as previously reported (Sribudiani et al., 2018). Alexa Fluor 488 AffiniPure Goat Anti-Mouse IgG (H + L) antibody (Yeasen, China) was used to incubate with the embryos, and images were acquired by a fluorescence microscope (SMZ25, Nikon, Chiyoda, Japan). The absence or reduction of enteric neurons in the distal intestine was defined as an HSCR-like phenotype (Jiang et al., 2015; Gui et al., 2017). We counted the number of enteric neurons in the most distal six somite lengths from the anal pore.

### Protein–Protein Interaction Network Analysis

To explore the potential correlations of *DSCAM* and *BACE2* with members from the *RET* activation pathway, the *EDNRB* signaling pathway, transcription factors during ENS formation, and other known disease genes, we construct a network utilized a web-based interface GeneMANIA^2^ (Warde-Farley et al., 2010). The key genes of *RET* activation pathway (*RET*, *GDNF*, *GFRA1*, *GFRA2*, and *NRTN*), *EDNRB* signaling pathway (*EDNRB*, *EDN3*, and *ECE1*), two transcription factors (*SOX10* and *PHOX2B*), and three genome-wide association study (GWAS)-reported genes (*NRG1*, *SEMA3C*, and *SEMA3D*), together with *DSCAM* and *BACE2*, were analyzed using GeneMANIA.

### Statistical Analysis

Statistical analyses were performed with GraphPad Prism 8 (GraphPad Software). All results are expressed as the mean ± standard error. A two-sided unpaired Student *t* test was carried out for statistical comparison between two groups. *P* < 0.05 was considered statistically significant.

## Results

### Association Analysis

Among the 133 SNPs genotyped, nine were excluded due to poor genotyping success rate (<5%). The remaining 124 SNPs had a genotyping success rate >99% and conformed to HWE (*P* > 0.01). The genotyping success rate of all individuals was above 98%. The distributions of the allele frequencies of these SNPs in HSCR patients and controls are shown in Supplementary Table 7. None of the 124 SNPs passed quality control and reached the study-level significance (*P* < 0.00038). Twelve intronic SNPs of *DSCAM* showed suggestive association, with a *P* < 0.05. None of the 19 *BACE2* SNPs met suggestive significance level (*P* < 0.05; Table 1 and Supplementary Table 7).

**TABLE 1:** Association of *DSCAM* SNPs with HSCR risk in 420 unrelated sporadic HSCR patients and 1,665 controls.

SNP	Position	Gene	Functional annotation	Reference allele	RAF		*P*-value	OR (95% CI)
					Case	Control		
rs430255	41,496,605	*DSCAM*	Intronic	G	0.862	0.821	0.0052	1.36 (1.10–1.68)
rs2837756	41,995,874	*DSCAM*	Intronic	G	0.438	0.389	0.0091	1.23 (1.05–1.43)
rs2837770	42,034,352	*DSCAM*	Intronic	G	0.575	0.560	0.42	1.07 (0.91–1.24)
rs8134673	42,048,311	*DSCAM*	Intronic	G	0.581	0.567	0.45	1.06 (0.91–1.24)

*The listed SNPs include two reported DSCAM SNPs and two most associated DSCAM SNPs. SNP, single-nucleotide polymorphism; RAF, risk allele frequency; OR, odds ratio; HSCR, Hirschsprung disease.*

**FIGURE 1:**
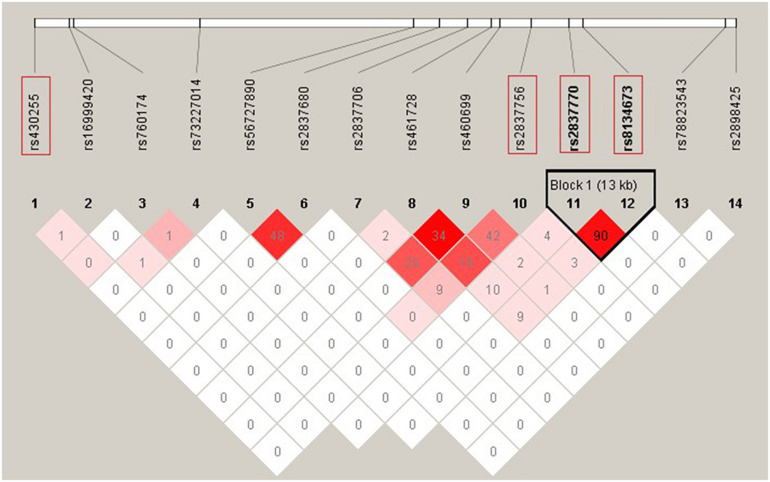
Linkage disequilibrium (LD) pattern of 12 *DSCAM* single-nucleotide polymorphisms (SNPs) with *P* < 0.05 in association analysis and two previous reported SNPs. The plot was constructed using the program Haploview, and *r*^2^ values (×100) between SNPs are shown in the diamonds. *r*^2^ values were calculated using data of 420 HSCR patients and 1,663 controls from the current study. *r*^2^ values of 1 represent complete LD, *r*^2^ values greater than 0.8 represent strong evidence of LD, *r*^2^ values of 0.2–0.8 represent moderate LD, and *r*^2^ values less than 0.2 represent low LD. Haplotype blocks were determined using the confidence interval method. Two SNPs (rs2837770 and rs8134673) reported associations with isolated HSCR in previous study, and the two most associated SNPs (rs430255 and rs2837756) are highlighted in the red rectangles.

Two SNPs (rs2837770 and rs8134673) that were reported to be associated with isolated HSCR showed no significant association in the current sample (*P* > 0.05). These two SNPs were in high LD (*r*^2^ = 0.90, Figure 1). The frequencies of associated allele rs2837770 G and rs8134673 G were both 0.54 in Chinese control samples of a previous study, which was similar to the frequencies in the current controls (rs2837770 G, 0.560; rs8134673 G, 0.567; Table 1). The frequencies of rs2837770 G (0.575) and rs8134673 G (0.581) were higher in the current cases than in controls, which showed the same effect direction as a previous study.

Rs430255 (*P*_*Addtive*_ = 0.0052, *OR* = 1.36, 95% CI: 1.10–1.68) and rs2837756 (*P*_*Addtive*_ = 0.0091, *OR* = 1.23, 95% CI: 1.05–1.43) showed the most remarkable association with a *P* < 0.01. Rs430255 and rs2837756 are located in intron 19 and intron 3 of *DSCAM*. Functional annotation revealed that rs430255, rs2837756, and SNPs in high LD with rs2837756 all altered the sequences of multiple transcription factor binding motifs (Supplementary Tables 8,9).

We also performed association analysis stratified by the segment length of aganglionosis. Both rs430255 (*P*_*Addtive*_ = 0.0088, *OR* = 1.38, 95% CI: 1.08–1.76) and rs2837756 (*P*_*Addtive*_ = 0.0021, *OR* = 1.31, 95% CI: 1.10–1.55) showed a slightly stronger association with S-HSCR than with the total cases. However, these two SNPs were not associated with susceptibility to L-HSCR and TCA. The two reported SNPs (rs2837770 and rs8134673) showed no association in the stratification analysis (Table 2 and Supplementary Table 10).

**TABLE 2:** Stratification analysis of *DSCAM* SNPs in 420 unrelated sporadic HSCR patients and 1,665 controls.

SNP	Reference allele	Reference allele frequency				S-HSCR vs. control		L-HSCR vs. control		S-HSCR vs. control	
		Control	S-HSCR	L-HSCR	TCA	*P*-value	OR (95% CI)	*P*-value	OR (95% CI)	*P*-value	OR (95% CI)
rs430255	G	0.821	0.864	0.853	0.859	0.0088	1.38 (1.08–1.76)	0.37	1.27 (0.75–2.14)	0.39	1.33 (0.70–2.52)
rs2837756	G	0.389	0.454	0.388	0.385	0.0021	1.31 (1.10–1.55)	0.99	1.00 (0.68–1.46)	0.94	0.98 (0.62–1.56)
rs2837770	G	0.560	0.579	0.543	0.590	0.36	1.08 (0.91–1.28)	0.73	0.94 (0.65–1.36)	0.59	1.13 (0.72–1.79)
rs8134673	G	0.567	0.587	0.535	0.603	0.35	1.09 (0.91–1.29)	0.49	0.88 (0.61–1.27)	0.53	1.16 (0.73–1.83)

*All the 420 patients consisted of 323 S-HSCR, 58 L-HSCR, and 39 TCA patients. The results of two reported DSCAM SNPs and two most associated DSCAM SNPs are shown. SNP, single-nucleotide polymorphism; RAF, risk allele frequency; OR, odds ratio; HSCR, Hirschsprung disease; TCA, total colonic aganglionosis.*

### Expression Pattern of *dscama*, *dscamb*, and *bace2* in Developing Embryos of Zebrafish

Our association analysis suggested a role for *DSCAM* in the susceptibility of HSCR. However, we could not exclude *BACE2* as a strong susceptibility gene for three reasons. First, the associated SNPs in *DSCAM* region might have a long-range regulation effect on *BACE2*, which is adjacent to *DSCAM*. Second, rare variants were associated with HSCR risk, which was not assessed in our study. Last, *BACE2* has a role in enteric neuron function (Fattahi et al., 2016; Tang et al., 2018). Therefore, we further explored the possible role of both *DSCAM* and *BACE2* in the development of ENS using zebrafish.

**FIGURE 2:**
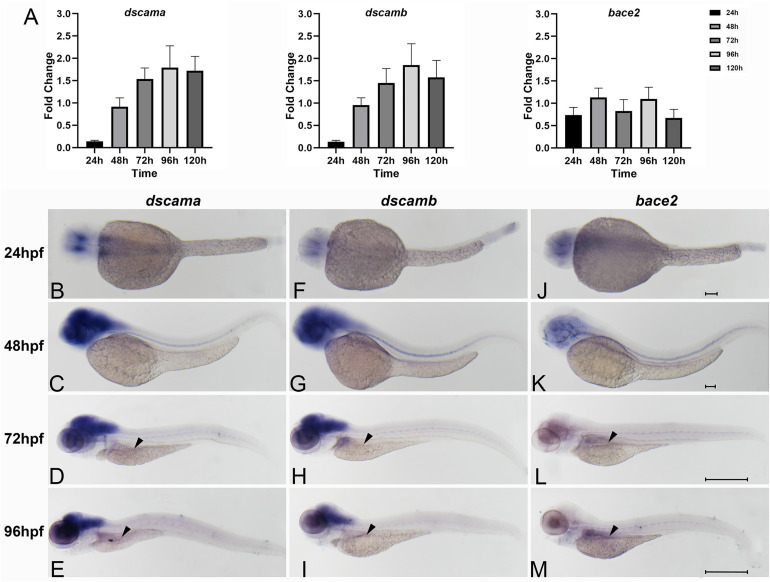
Spatiotemporal expression of zebrafish of *dscama*, *dscamb*, and *bace2* during early embryogenesis. **(A)** qRT-PCR analysis of relative expression levels of *dscama*, *dscamb*, and *bace2* in zebrafish embryos from 24 to 120 hpf. **(B–M)** Whole-mount *in situ* hybridization in wild-type embryos for *dscama* **(B–E)**, *dscamb* **(F–I)**, and *bace2* **(J–M)**. Strong expression of *dscama* **(B–E)** and *dscamb* **(F–I)** was observed in the brain and central nervous system after 24 hpf, and a weakly positive expression signal was observed in the intestinal bulb at 72 and 96 hpf (black arrowheads). **(J–K)** *bace2* expression in the neural crest cells and pigment epithelial cells of the retina at 24 and 48 hpf. A relatively strong expression signal of *bace2* was observed in the intestinal primordium after 72 hpf indicated by black arrowheads **(L,M)**. Scale bar for 24 and 48-hpf embryos is 100 μm, and that for 72 and 96-hpf embryos is 500 μm.

We investigated the expression levels of the zebrafish orthologs during early development. A search in the Ensemble database revealed that there were two orthologs of *DSCAM*, namely, *dscama* and *dscamb*, which showed high sequence similarity with the human orthologs (84.9 and 79.5% homology, respectively). Similarly, the sequence of *bace2* has 63.1% sequence homology to human *BACE2*. The qRT-PCR results showed that the expression patterns of *dscama* and *dscamb* were similar in the early stages during zebrafish development. The lowest expression levels of the two genes were observed 24 hpf, which increased gradually from 24 to 96 hpf. The expression level of *bace2* was lower than that of *dscama* and *dscamb* in the embryos of zebrafish (Figure 2A).

We further determined the spatiotemporal expression patterns of the three genes with WISH analysis using antisense nucleic acid probes. The results showed that *dscama* mRNA was strongly expressed in the midbrain, telencephalon, and diencephalon. At 24 and 48 hpf, widespread *dscama* expression was observed in the central nervous system (CNS), the hindbrain and spinal cord (Figures 2B,C). The expression pattern of *dscamb* mRNA was similar to that of *dscama* (Figures 2F,G). It was known that neural precursors differentiated and began to extend axons and dendrites in these regions (Yimlamai et al., 2005). Of note, *dscama* and *dscamb* began to be expressed in the gut tube in zebrafish larvae at 72 hpf (Figures 2D,E,H,I). These results suggested that *dscams* might play a role in the development of CNS and ENS. We observed that *bace2* began to be expressed in the neural crest cells and pigment epithelial cells of the retina from 48 hpf (Figures 2J,K). An expression signal for *bace2* was detected in the intestinal primordium at 72 hpf, and the signal was significantly enhanced at 96 hpf (Figures 2L,M). The results indicated that *bace2* might have a more significant effect in the intestine than *dscams.*

### Overexpression of *DSCAM* and *bace2* Caused Developmental Defects in the Enteric Nervous System

**FIGURE 3:**
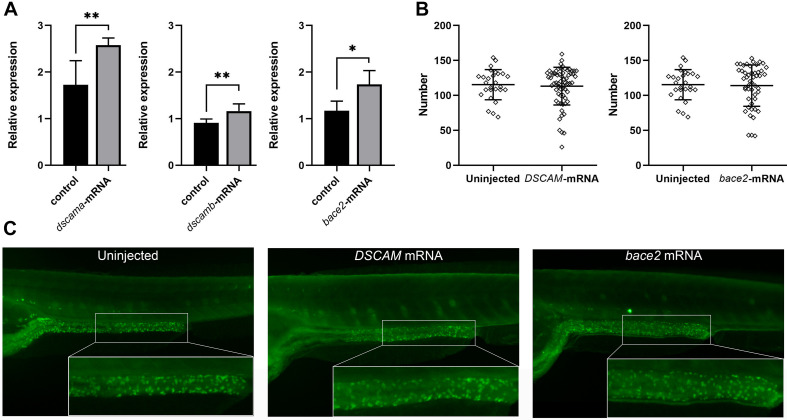
Phenotypes of zebrafish embryos with overexpression of *DSCAM* and *bace2*. Zebrafish embryos were injected with 100 pg of *DSCAM* mRNA per embryo and 100 pg of *bace2* mRNA per embryo, respectively. **(A)** The expression levels of *dscama*, *dscamb*, and *bace2* of mRNA injected embryos were analyzed by qRT-PCR at 48 hpf (^∗^*P* < 0.05, ^∗∗^*P* < 0.01). **(B)** No significant difference was found in the neuron numbers of the last six somite lengths of gut between control and mRNA injected embryos at 5 dpf. The HuC/D antibody was used to stain the enteric nervous system (ENS) neurons, and the neuron numbers in the last six somite lengths of gut were counted. Embryos injected with *DSCAM* mRNA, *n* = 67. Embryos injected with *bace2* RNA, *n* = 53. Control embryos, *n* = 28. **(C)** The overexpression of *DSCAM* and *bace2* caused no obvious defect of enteric neurons in the hindgut.

DS HSCR is caused by the presence of three copies of total or partial chromosome 21. It is assumed that chr 21 transcripts are overexpressed by about 50% in cells with an extra copy of this chromosome. Recent studies have demonstrated an increased transcript level of the three-copy genes for a subset of them (Lockstone et al., 2007; Prandini et al., 2007; Letourneau et al., 2014; Olmos-Serrano et al., 2016). We, therefore, injected h*DSCAM* and *bace2* mRNA into the embryos of zebrafish to test the effect of gene overexpression on the development of ENS. The qRT-PCR analysis showed that the expression of target genes was markedly increased (Figure 3A). Embryos injected with both h*DSCAM* and *bace2* mRNA at 5 dpf exhibited no gross morphological defects and no reduction of enteric neurons in the distal intestine as compared with control embryos (*P* > 0.05; Figures 3B,C).

### Knockdown of *dscama*, *dscamb*, and *bace2* Resulted in Reduction of Enteric Neurons in Zebrafish

**FIGURE 4:**
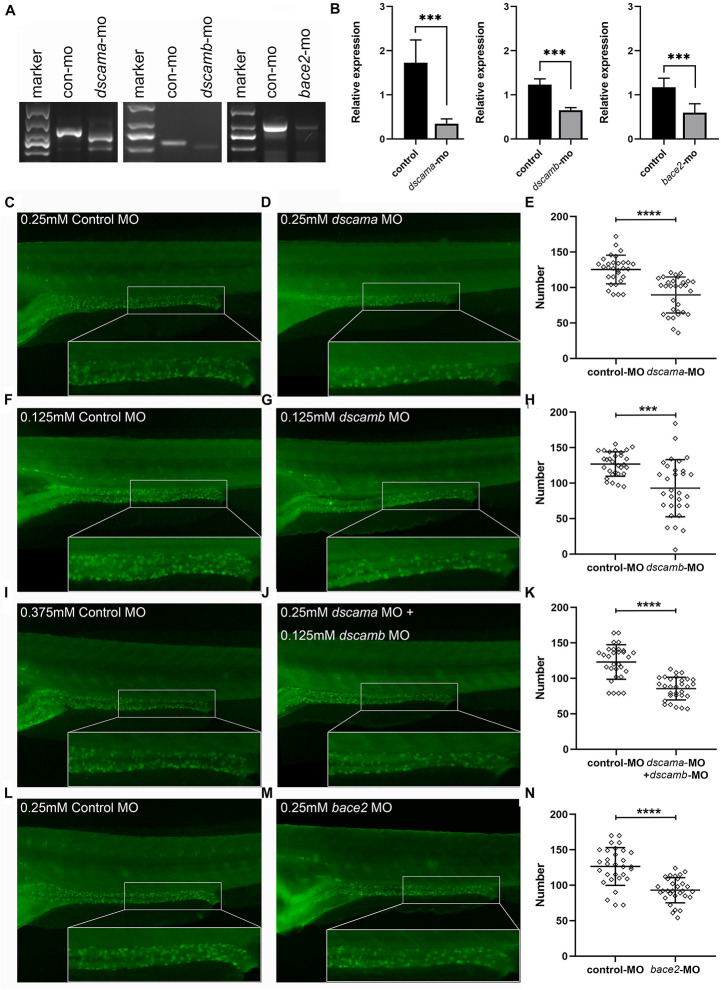
Phenotypes of *dscama*, *dscamb*, and *bace2* knockdown zebrafish. Embryos at 5 dpf were stained with HuC/D antibody to observe the intestinal neurons. The neuron numbers in the last six somite lengths of gut were counted. **(A)** RT-PCR confirmation of splice-blocking morpholino oligonucleotide (SBMO) knockdown in embryos at 48 hpf. In *dscama* morphants, two small fragments were observed. In *dscamb* morphants, a shorter fragment was produced, and the amount of PCR products decreased. The length of PCR products from mRNA of *bace2* morphants was same as control MO-injected embryos, but the amount of PCR product decreased obviously. **(B)** qRT-PCR analysis of *dscama*, *dscamb*, and *bace2* mRNA expression after morpholino injection. The target gene expression levels of SBMO-injected embryos were remarkably reduced compared with the controls. **(C–E)** Compared with the 0.25 mM control MO injection, 0.25 mM *dscama* MO injection resulted in a decrease in the density of enteric neurons. **(F–H)** Embryos injected with 0.125 mM of control MO and 0.125 mM of *dscamb* MO. **(I–K)** Embryos injected with 0.375 mM of control MO and 0.25 mM of *dscama* MO + 0.125 mM of *dscamb* MO. **(L–N)** Embryos injected with 0.25 mM of control MO and 0.25 mM of *bace2* MO. ^∗∗∗^*P* < 0.001, ^*⁣*⁣**^P < 0.0001.

*DSCAM* and *BACE2* were not found to be up-regulated in tissues of DS patients or mouse model in previous studies (Lockstone et al., 2007; Prandini et al., 2007; Letourneau et al., 2014; Olmos-Serrano et al., 2016). Additionally, the deficiency of other known HSCR genes accounted for the disease risk (Emison et al., 2005; Amiel et al., 2008; Garcia-Barcelo et al., 2009; Jiang et al., 2015; Gui et al., 2017; Porokuokka et al., 2019). Therefore, we used splicing MO to interfere with the expression of *dscama*, *dscamb*, and *bace2* in zebrafish embryos to explore the effect of loss of function of the two genes in ENS development (Figure 4). The RT-PCR results confirmed the knockdown of target genes by SBMO, and the expression levels of target genes were significantly reduced in the splicing-MO embryos (Figures 4A,B). Both *dscama* and *dscamb* MO injection caused obvious morphological defects in a proportion of embryos, usually manifested as the overall shortening of the embryos and multiple tissue disorders, the most common of which was the shrinking brain and/or curling tail (Supplementary Figure 1). When the two MOs were co-injected, the proportion of abnormal embryos increased and the morphological defects became more serious. Importantly, immunostaining with antibodies against a neuronal marker, HuC/D, showed that the density of enteric neurons was significantly reduced in the distal intestine of *dscama* MO, *dscamb* MO, and co-injection morphants at 5 dpf as compared with control MO embryos.

Since increasing the amount of morpholino injection led to a higher mortality rate of larvae, for further enteric neurons counting, we chose a dose that could reduce the number of enteric neurons but cause the minimum death rate. The abnormality rate was 13.1% (*n* = 84) for 0.25 mM of *dscama* MO injection, while it was 60.0% (*n* = 60) for 0.25 mM of *dscamb* MO injection. We decreased the concentration of *dscamb* MO to 0.125 mM, and the dysmorphic rate became 13.41% (*n* = 82) but was still effective. Immunostaining results showed that the average number of enteric neurons was 89.53 ± 25.41 in the last six somite lengths of 0.25 mM of *dscama* MO morphants and 92.83 ± 40.20 in 0.125 mM of *dscamb* MO morphants. Both were significantly lower than that in embryos injected with control MO (125.4 ± 20.19 for 0.25 mM of control MO, *P* < 0.0001; 126.90 ± 17.18 for 0.125 mM of control MO, *P* = 0.0001, Figures 4C–H). When 4.2 ng/embryo *dscama* MO and 0.125 mM of *dscamb* MO were co-injected, the reduction of enteric neuron was more significant (85.53 ± 16.02; *n* = 95) as compared with embryos injected with 0.375 mM of control MO (123.1 ± 24.28; *P* < 0.0001, Figures 4I–K).

The *bace2* morphants injected with 0.25 mM of MO appeared morphologically normal. However, immunostaining analysis showed that the number of enteric neurons was significantly reduced in the distal intestine of *bace2* MO morphants 5 dpf as compared with control MO embryos (93.00 ± 17.70 vs. 126.6 ± 26.60; *P* < 0.0001, Figures 4L–N).

### Protein–Protein Interaction Network of *DSCAM* and *BACE2*

**FIGURE 5:**
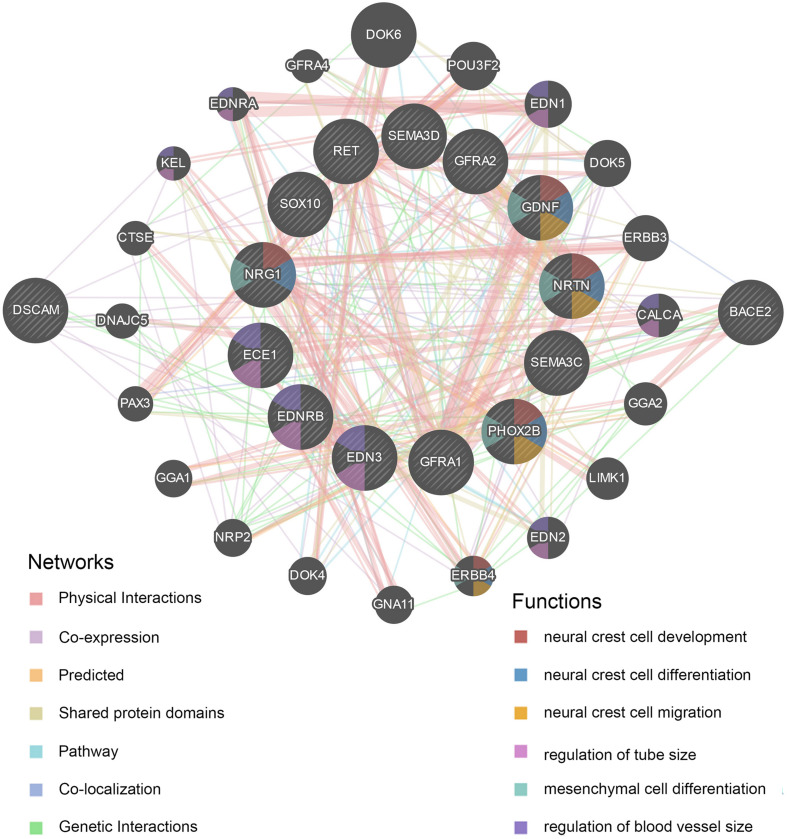
Protein–protein interaction (PPI) network of *DSCAM* and *BACE2*. The plot was constructed on GeneMANIA website. The correlations of *DSCAM* and *BACE2* with critical signaling pathway genes were illustrated. The 13 central nodes represent the critical susceptibility genes of HSCR. The surrounded 20 nodes represent genes highly relevant to the central nodes in terms of physical interaction, co-expression, prediction, pathway, co-localization, genetic interactions, and shared protein domains. The color of connecting lines between nodes represents the type of protein–protein interaction (PPI). The color of the node represents the possible functions of the genes.

A protein–protein interaction (PPI) network for the *DSCAM* and *BACE2* with critical signaling pathway genes was constructed, and the correlations were evaluated using the GeneMANIA database (Figure 5 and Supplementary Table 11). *DSCAM* was co-expressed with *GFRA1*, *NRTN*, *ERBB4*, *SOX10*, and *PAX3* and was correlated with *EDNRB*, *GFRA1*, and *SEMA3C* in terms of genetic interactions. *BACE2* was co-expressed with *NRG1*, *ERBB3*, and *PAX3* and had genetic interactions with *EDNRB*, *GFRA1*, *EDN2*, *EDN3*, *ERBB4*, *PAX3*, and *SEMA3C*. Further functional prediction revealed that these proteins showed correlations with neural crest cell development [false discovery rate (FDR) = 2.60 × 10^–6^), neural crest cell differentiation (FDR = 4.07 × 10^–6^), and neural crest cell migration (FDR = 6.12 × 10^–5^).

## Discussion

HSCR is a highly heritable disorder. Although many genetic variants were identified in recent years, they explained only a small proportion of HSCR heritability. In this study, we made an effort to clarify the contribution of *DSCAM* and *BACE2* in HSCR-associated critical region at chr 21. Our case–control analysis found that common variants of *DSCAM* showed suggestive association with HSCR susceptibility, while common variants of *BACE2* showed no association. Knockdown the expression of *DSCAM* and *BACE2* caused reduced numbers of enteric neurons in zebrafish. PPI analysis showed that both genes closely correlated with the critical genes involved in ENS development or underlying HSCR pathogenesis.

We did not find the two reported *DSCAM* SNPs to be associated with HSCR susceptibility, but we identified two novel associated SNPs. Notably, the effect direction of these two reported SNPs was consistent with previous study in Chinese population (Wang et al., 2018). These evidences supported that common variants of *DSCAM* conferred moderate risk to HSCR susceptibility. A previous study based on whole-genome sequence analysis in 443 cases and 493 controls identified rare variants in *BACE2* associated with HSCR (Tang et al., 2018). Our study found no association of common variants of *BACE2* with HSCR risk. We did not investigate association of rare variants in *BACE2* in the current study. Further studies are needed to investigate associations of both the common variants and rare ones in *BACE2* with risk to HSCR. Recent genome-wide association studies for HSCR risk did not report genome-wide significant signal at this locus, which suggested variants at this locus exerting a moderate influence on HSCR risk (Tang et al., 2012, 2018; Jiang et al., 2015). The relatively small sample size is the limitation of current study. Under the assumption of 0.0002 disease prevalence, the significance level of 0.05, and odds ratios of 1.3/1.6 for heterozygotes/risk homozygotes, 420 cases and 1,665 controls could achieve 52.5 and 86.3% statistical power for rs430255 [risk allele frequency (RAF) = 0.821] and rs2837756 (RAF = 0.389). Further increasing the sample size could unravel more HSCR-associated variants.

Both *DSCAM* and *BACE2* are functionally linked with the development of ENS. *DSCAM* has long been considered an attractive candidate gene for the increased incidence of HSCR in DS patients (Jannot et al., 2013). It encodes a member of the immunoglobulin superfamily that represents a class of neural cell adhesion molecules. *DSCAM* is widely expressed in the developing nervous system including the neural tube, spinal cord, and most neural crest-derived tissues (Yamakawa et al., 1998; Montesinos, 2014). It plays an important role in vertebrate neural development mediating homophile attraction in neuronal hierarchical targeting, and participating in the process of axon and dendrite self-avoidance and tiling (Hattori et al., 2008; Schmucker and Chen, 2009). In addition, *DSCAM* and deleted in colorectal carcinomas (*DCC*) are both receptors of *netrin-1*, which serves as an important axon guidance cue during neural development (Ly et al., 2008). Netrin-mediated guidance is related to the vertical migration of enteric neural crest-derived cells (ENCDCs), which derive submucosal and pancreatic plexus (Jiang et al., 2003; Ratcliffe et al., 2006). Deficiency of *Dcc* resulted in loss of submucosal ganglia in gut of mice model (Jiang et al., 2003). Our WISH results are consistent with prior research that *dscams* were highly expressed in zebrafish CNS, which might explain the underlying mechanisms of the multiple defects of *dscams* morphants (Yamakawa et al., 1998; Montesinos, 2014). The expression of *dscams* in developing gut of zebrafish and the abnormalities of ENS caused by dysfunction of *dscams* indicated their involvement in the pathogenesis of HSCR. Together with previous findings, our results highlighted the importance of *netrin-1*/*DCC*/*DSCAM* pathway in the ENS development and HSCR pathology.

BACE2 has beta-secretase cleavage activity against amyloid precursor protein (APP). Deposition in the brain of the 39- to 43-amino acid APP is a hallmark of Alzheimer disease (AD), a frequent complication of DS patients after the age of 30 years. BACE2 has been considered an important enzyme for AD pathogenesis or therapy (Wang et al., 2019). The accumulation of Aβ in the brain induces neuronal apoptosis, which is a critical step in the etiology of AD (Wang et al., 2019). BACE2 could protect the ENS neurons from undergoing apoptosis by properly processing APP and preventing the Aβ accumulation, indicating that the BACE1–APP–BACE2 pathway might be a causal pathway in the pathogenesis of HSCR (Tang et al., 2018). *BACE2* expression in the brain is rather weak. We observed that *bace2* morphants showed no gross abnormality, which was consistent with previous studies that *Bace2*-null mice showed no abnormality (Dominguez et al., 2005). The detection of *bace2* expression in gut tube indicated a causal role of this gene in ENS development. Knockdown *bace2* could cause reduced enteric neuron numbers in the hindgut of zebrafish, which further supported *BACE2* as a HSCR risk gene.

In zebrafish embryos, the enteric neurons occupy the middle and distal intestine at 4–5 dpf, and a few enteric neurons can be observed around the proximal gut at this time (Olsson et al., 2008). The number of HuC/D-positive cells in the proximal gut increased gradually, and the enteric cells were commonly seen in the anterior half of the intestinal bulb after 9 dpf (Olsson et al., 2008). MO knockdown of *dscams* and *bace2* caused the reduction of neurons in the gut, but not aganglionosis in the distal intestine. Considering that if *DSCAM* and *BACE2* have an effect in HSCR pathogenesis, deficiency of the two genes might increase predisposition for the disease. Therefore, in a future study, it would be interesting to investigate what happens if these two genes are knocked down in a genetic compromised background, such as when *RET* expression is compromised.

Although the occurrence of HSCR in DS patients is 40-fold more common than in the general population of newborns, only about 0.8% of individuals with DS have HSCR (Arnold et al., 2009). Thus, the existence of trisomy 21 does not invariably lead to HSCR. It has generally been assumed that a 1.5-fold increase in gene dosage produces the phenotypes of DS and DS-associated syndrome (Arnold et al., 2009; Schill et al., 2019). Unexpectedly, our results showed that loss of function of the two genes resulted in reduced numbers of neurons in the hindgut. One explanation for this curious finding may be that these two genes may not account for the contribution of trisomy 21 to HSCR risk. A recent study found that the ENS defect in two DS mouse models could not be rescued by normalizing copy number for *Dscam*, challenging that *DSCAM* explains increased HSCR risk in patients with DS (Schill et al., 2019). However, it is reasonable that *DSCAM* plays a role in the pathogenesis of sporadic HSCR, since *DSCAM* variants were found to be associated with sporadic HSCR risk.

Some of the disease-associated common variants could be simply hidden below the threshold of significance for the relatively small sample size in HSCR association studies. Genetic clues in combination with animal models might help to detect more such susceptibility genes with smaller effect size. Our study provided further evidence in support of the contribution of common variants in HSCR-associated critical region at chromosome 21 to sporadic HSCR susceptibility, and it demonstrated the causal role of *DSCAM* and *BACE2* in defects of ENS. These findings might facilitate disentangling of the complex contribution of this critical disease-associated region to HSCR pathogenesis.

## Data Availability Statement

The datasets presented in this study can be found in online repositories. The names of the repository/repositories and accession number(s) can be found in the article/ Supplementary Material.

## Ethics Statement

The studies involving human participants were reviewed and approved by the Institutional Review Board of Xinhua Hospital affiliated to the Shanghai Jiao Tong University School of Medicine. Written informed consent to participate in this study was provided by the participants’ legal guardian/next of kin. The animal study was reviewed and approved by the Animal care and Use Committee of Xinhua Hospital.

## Author Contributions

XC and WC conceived the study. Y-JL, W-WY, X-XY, H-LS, M-RB, and M-MC conducted the experiment. W-JW, B-LG, and JW collected the samples. Y-JL and XC participated in the data analysis and figures preparation, and drafted the manuscript. All authors read and approved the final manuscript.

## Conflict of Interest

The authors declare that the research was conducted in the absence of any commercial or financial relationships that could be construed as a potential conflict of interest.

## Footnotes

1 http://gvs.gs.washington.edu/GVS150/

2 http://www.genemania.org
