# Supplementary material for: Association Analysis of Variants of DSCAM and BACE2 With Hirschsprung Disease Susceptibility in Han Chinese and Functional Evaluation in Zebrafish
Source: Front Cell Dev Biol. 2021 May 31;9:641152. doi: 10.3389/fcell.2021.641152 (PMC8201997; doi:10.3389/fcell.2021.641152)
Supplement: Supplementary file 1 [file Data_Sheet_1.docx]

**Supplementary Figure and Tables**

**
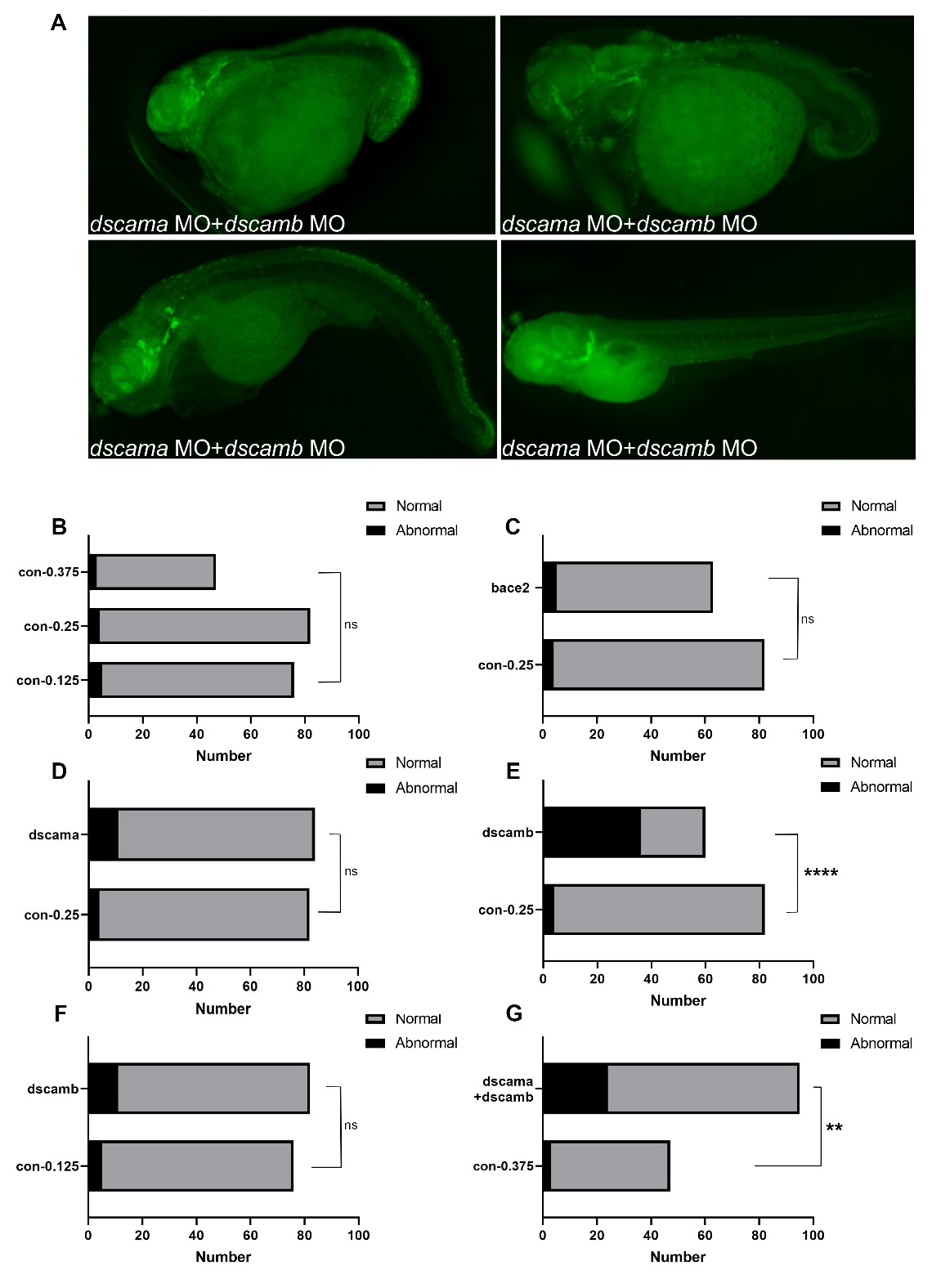
­­**

**Supplementary Figure 1.** Morphological abnormalities in morphants **(A)** The typical manifestations of morphological abnormalities in a proportion of morphants included the overall shortening of the embryos, the shrinking brain, enlarging heart and curling tail; **(B)** The percentages of morphological abnormalities had no difference among three groups of embryos injected with different amounts of control MO (0.375mM control MO: 3/47=6.38%, 0.25mM control MO: 4/82=4.88%, 0.125mM control MO: 5/76=6.58%; *P* > 0.05); **(C)** The percentages of morphological abnormalities had difference between morphants injected with 0.25mM *bace2* MO and control group with 0.25mM control MO (5/63=7.94% *vs.* 4/82=4.88%; *P* > 0.05); **(D)** The percentages of morphological abnormalities had difference between embryos injected with 0.25mM *dscama* and control group with 0.25mM control MO (11/84 = 13.10% *vs.* 4/82=4.88%, *P* > 0.05); **(E)** The *dscamb* morphants injected with 0.25mM MO show a significantly higher prevalence of morphological abnormalities compared with controls (36/60=60% *vs.* 4/82=4.88%; *P* < 0.0001); **(F)** The percentages of morphological abnormalities had difference between embryos injected with 0.125mM *dscama* and control group with 0.25mM control MO (11/82=13.41% *vs.* 5/76=6.58%; *P* > 0.05); **(G)** The morphants co-injected with 0.25mM *dscama* MO and 0.125mM *dscamb* MO showed a significantly higher prevalence of morphological abnormalities compared with controls (24/95=25.26% *vs.* 3/47=6.38%; *P*=0.0063).

**Supplementary Table 1.** Primers for qRT-PCR evaluation of gene expression levels in wild type zebrafish embryos.

| Gene | Forward Primers (5’-3’) | Reverse Primers (5’-3’) |  | Product Size |
| --- | --- | --- | --- | --- |
| *18-s* | TCGCTAGTTGGCATCGTTTATG | CGGAGGTTCGAAGACGATCA |  | 117bp |
| *dscama* | AGCAATCACATCCCAGAGCA | ACCACCACCTCATACTCAGC |  | 210bp |
| *dscamb* | TGCAGGCAGTTATGTGTGTG | TCTCCGCATTCCTGTACCAG |  | 197bp |
| *bace2* | AGCACATCCCTACATCACACA | TGCAAGTCCAAGAATCCCCT |  | 238bp |

**Supplementary Table 2.** Primers for RNA probes used in whole mount in situ hybridization.

| Gene | Forward Primers (5’-3’) | Reverse Primers (5’-3’) | Product Size |
| --- | --- | --- | --- |
| *dscama* | GAGGAGAGCTACAACGTCC | TCTGCCGTTATGCTGTGAA | 1199bp |
| *dscamb* | AACCTGTGGATTCTGCTCCG | CGAGCAAAGCACTGATACGC | 508bp |
| *bace2* | AATTCAACGCGTCTGTGCAG | TTGCGGCAATGTTGATGGTG | 414bp |

**Supplementary Table 3.** Sequences of morpholino oligo used in knock-down assays.

| Gene | Morpholino oligo sequence (5’-3’) |
| --- | --- |
| control-MO | CCTCTTACCTCAGTTACAATTTATA |
| *dscama*-MO | AAAGATCCTGAAATGCTCACCGGCC |
| *dscamb*-MO | CCAACACAAAGGCCGCTCACCTGAT |
| *bace2*-MO | TGGAACTGAGCAAACCATCATAACA |

**Supplementary Table 4.** Primers used to verify the efficiency of morpholino injection using gel electrophoresis of PCR products.

| Gene | Forward Primers (5’-3’) | Reverse Primers (5’-3’) | Product Size |
| --- | --- | --- | --- |
| *dscama* | AAGAGGTAGTGTTCGCCAGC | CGTGTAGCGATGTCTCGTCA | 518bp |
| *dscamb* | TTCTGGATGTGCAGTCGGAG | CACATAACTGCCTGCATCGC | 329bp |
| *bace2* | GCTCTACGGGCTACTGCTAC | GTCCAAGAATCCCCTGCCAA | 554bp |

**Supplementary Table 5.** Primers used to verify the efficiency of morpholino injection using qRT-PCR.

| Genes | Forward Primers (5’-3’) | Reverse Primers (5’-3’) | Product Size |
| --- | --- | --- | --- |
| *18-s* | TCGCTAGTTGGCATCGTTTATG | CGGAGGTTCGAAGACGATCA | 117bp |
| *dscama* | GCACAGCGGAAAATCCTTCA | ACGGTGTCTCTCTCCCATGA | 190bp |
| *dscamb* | TGGGAGAAAGACACAGTCTCA | CTGATAAAAGCAGTCGGGCAC | 178bp |
| *bace2* | GCAGCAGCACATCCCTACAT | TTGCGGCAATGTTGATGGTG | 181bp |

**Supplementary Table 6.** Primers used to verify the efficiency of mRNA injection using qRT-PCR.

| Genes | Forward Primers (5’-3’) | Reverse Primers (5’-3’) | Product Size |
| --- | --- | --- | --- |
| *18-s* | TCGCTAGTTGGCATCGTTTATG | CGGAGGTTCGAAGACGATCA | 117bp |
| *dscama* | GCACAGCGGAAAATCCTTCA | ACGGTGTCTCTCTCCCATGA | 190bp |
| *dscamb* | TATGCCAGCCAGTGGACCCT | TAAGTGGAGGAGGCGCTTTCT | 197bp |
| *bace2* | GCAGCAGCACATCCCTACAT | TTGCGGCAATGTTGATGGTG | 181bp |

**Supplementary Table 7.** Association results of 133 tag SNPs in 420 unrelated sporadic HSCR patients and 1,665 controls.

| SNP | BP | GENE | Functional annotation | Reference allele | Alternative allele | RAF | |  | Cases *vs* control | |
| --- | --- | --- | --- | --- | --- | --- | --- | --- | --- | --- |
|  |  |  |  |  |  | Cases | Controls |  | P | OR 95%CI |
| rs62235573 | 41,388,084 | *DSCAM* | intronic | A | G | 0.898 | 0.887 |  | 0.3819 | 1.12( 0.87- 1.43) |
| rs9975444 | 41,392,131 | *DSCAM* | intronic | A | C | 0.956 | 0.946 |  | 0.2446 | 1.24( 0.86- 1.78) |
| rs9975710 | 41,400,858 | *DSCAM* | intronic | G | A | 0.094 | 0.094 |  | 0.9962 | 1( 0.77- 1.30) |
| rs9981861 | 41,415,044 | *DSCAM* | intronic | A | G | 0.826 | 0.811 |  | 0.3213 | 1.11( 0.91- 1.35) |
| rs2142123 | 41,418,208 | *DSCAM* | intronic | G | A | 0.866 | 0.852 |  | 0.3203 | 1.12( 0.90- 1.39) |
| rs2837402 | 41,428,836 | *DSCAM* | intronic | G | A | 0.075 | 0.068 |  | 0.5067 | 1.1( 0.83- 1.47) |
| rs11701316 | 41,435,240 | *DSCAM* | intronic | A | G | 0.855 | 0.843 |  | 0.3805 | 1.1( 0.89- 1.36) |
| rs7276767 | 41,436,601 | *DSCAM* | intronic | A | G | 0.121 | 0.102 |  | 0.1113 | 1.21( 0.96- 1.54) |
| rs373996 | 41,448,911 | *DSCAM* | intronic | A | G | 0.254 | 0.250 |  | 0.838 | 1.02( 0.86- 1.21) |
| rs75667921 | 41,450,390 | *DSCAM* | intronic | G | A | 0.135 | 0.132 |  | 0.8171 | 1.03( 0.82- 1.28) |
| rs2837426 | 41,454,734 | *DSCAM* | intronic | G | A | 0.283 | 0.274 |  | 0.5716 | 1.05( 0.89- 1.24) |
| rs1734936 | 41,488,565 | *DSCAM* | intronic | A | G | 0.372 | 0.372 |  | 0.9735 | 1( 0.86- 1.17) |
| rs2837430 | 41,489,655 | *DSCAM* | intronic | G | A | 0.647 | 0.628 |  | 0.3216 | 1.08( 0.92- 1.27) |
| rs17828614 | 41,492,270 | *DSCAM* | intronic | G | A | 0.936 | 0.923 |  | 0.2038 | 1.22( 0.90- 1.65) |
| rs430255 | 41,496,605 | *DSCAM* | intronic | G | A | 0.862 | 0.821 |  | 0.0052 | 1.36( 1.10- 1.68) |
| rs2837445 | 41,505,308 | *DSCAM* | intronic | G | A | 0.261 | 0.251 |  | 0.5406 | 1.06( 0.89- 1.26) |
| rs57550834 | 41,512,872 | *DSCAM* | intronic | A | G | 0.301 | 0.285 |  | 0.3541 | 1.08( 0.92- 1.28) |
| rs2837455 | 41,518,280 | *DSCAM* | intronic | G | A | 0.374 | 0.370 |  | 0.8369 | 1.02( 0.87- 1.19) |
| rs75557627 | 41,522,683 | *DSCAM* | intronic | A | G | 0.883 | 0.876 |  | 0.5612 | 1.07( 0.85- 1.36) |
| rs73225241 | 41,523,288 | *DSCAM* | intronic | G | A | 0.949 | 0.938 |  | 0.2561 | 1.22( 0.87- 1.71) |
| rs73362149 | 41,524,468 | *DSCAM* | intronic | A | C | 0.196 | 0.189 |  | 0.6189 | 1.05( 0.87- 1.27) |
| rs8132673 | 41,525,276 | *DSCAM* | intronic | C | A | 0.719 | 0.718 |  | 0.969 | 1( 0.85- 1.19) |
| rs2837464 | 41,529,188 | *DSCAM* | intronic | A | G | 0.349 | 0.325 |  | 0.1977 | 1.11( 0.95- 1.30) |
| rs7275239 | 41,531,337 | *DSCAM* | intronic | A | G | 0.741 | 0.723 |  | 0.2867 | 1.1( 0.92- 1.31) |
| rs2837466 | 41,531,472 | *DSCAM* | intronic | G | A | 0.300 | 0.290 |  | 0.5923 | 1.05( 0.89- 1.24) |
| rs16999420 | 41,531,893 | *DSCAM* | intronic | A | G | 0.958 | 0.940 |  | 0.0383 | 1.47( 1.02- 2.12) |
| rs7280615 | 41,532,663 | *DSCAM* | intronic | G | A | 0.280 | 0.261 |  | 0.2818 | 1.1( 0.93- 1.30) |
| rs8130234 | 41,536,157 | *DSCAM* | intronic | G | A | 0.711 | 0.685 |  | 0.1543 | 1.13( 0.96- 1.33) |
| rs760174 | 41,536,215 | *DSCAM* | intronic | A | G | 0.357 | 0.319 |  | 0.0348 | 1.19( 1.01- 1.39) |
| rs7278294 | 41,547,881 | *DSCAM* | intronic | A | G | 0.653 | 0.646 |  | 0.7163 | 1.03( 0.88- 1.21) |
| rs78800938 | 41,555,782 | *DSCAM* | intronic | A | C | 0.124 | 0.111 |  | 0.3013 | 1.13( 0.90- 1.43) |
| rs8127634 | 41,566,809 | *DSCAM* | intronic | A | G | 0.301 | 0.300 |  | 0.9274 | 1.01( 0.85- 1.19) |
| rs2837499 | 41,577,819 | *DSCAM* | intronic | G | A | 0.635 | 0.623 |  | 0.4995 | 1.06( 0.90- 1.24) |
| rs76045869 | 41,639,831 | *DSCAM* | intronic | G | A | 0.807 | 0.785 |  | 0.1592 | 1.15( 0.95- 1.39) |
| rs717416 | 41,649,097 | *DSCAM* | intronic | A | G | 0.656 | 0.639 |  | 0.3717 | 1.08( 0.92- 1.26) |
| rs73227014 | 41,662,855 | *DSCAM* | intronic | C | A | 0.104 | 0.081 |  | 0.0402 | 1.3( 1.01- 1.68) |
| rs8133535 | 41,664,277 | *DSCAM* | intronic | G | A | 0.149 | 0.145 |  | 0.7609 | 1.03( 0.84- 1.28) |
| rs2837565 | 41,672,532 | *DSCAM* | intronic | G | A | 0.375 | 0.355 |  | 0.2719 | 1.09( 0.93- 1.28) |
| rs34336407 | 41,684,090 | *DSCAM* | synonymous | A | G | 0.115 | 0.100 |  | 0.2252 | 1.16( 0.91- 1.48) |
| rs2026272 | 41,686,139 | *DSCAM* | intronic | C | A | 0.382 | 0.368 |  | 0.4537 | 1.06( 0.91- 1.24) |
| rs1571717 | 41,698,322 | *DSCAM* | intronic | G | A | 0.432 | 0.425 |  | 0.7002 | 1.03( 0.88- 1.20) |
| rs2205081 | 41,708,994 | *DSCAM* | intronic | A | G | 0.477 | 0.474 |  | 0.8435 | 1.02( 0.87- 1.18) |
| rs2837583 | 41,739,992 | *DSCAM* | intronic | A | G | 0.227 | 0.223 |  | 0.7626 | 1.03( 0.86- 1.23) |
| rs2210268 | 41,763,258 | *DSCAM* | intronic | A | C | 0.789 | 0.779 |  | 0.5221 | 1.06( 0.88- 1.28) |
| rs2837611 | 41,764,736 | *DSCAM* | intronic | A | C | 0.562 | 0.527 |  | 0.0717 | 1.15( 0.99- 1.34) |
| rs76750953 | 41,776,046 | *DSCAM* | intronic | A | G | 0.798 | 0.779 |  | 0.2344 | 1.12( 0.93- 1.35) |
| rs56342251 | 41,782,373 | *DSCAM* | intronic | A | G | 0.855 | 0.853 |  | 0.896 | 1.01( 0.82- 1.26) |
| rs1882755 | 41,786,287 | *DSCAM* | intronic | A | G | 0.201 | 0.193 |  | 0.6317 | 1.05( 0.87- 1.27) |
| rs1882757 | 41,790,020 | *DSCAM* | intronic | A | G | 0.406 | 0.388 |  | 0.3487 | 1.08( 0.92- 1.26) |
| rs2837620 | 41,792,388 | *DSCAM* | intronic | G | A | 0.593 | 0.583 |  | 0.5892 | 1.04( 0.89- 1.22) |
| rs8129283 | 41,805,008 | *DSCAM* | intronic | A | G | 0.107 | 0.092 |  | 0.1674 | 1.19( 0.93- 1.53) |
| rs10439672 | 41,813,285 | *DSCAM* | intronic | G | A | 0.554 | 0.542 |  | 0.5551 | 1.05( 0.90- 1.22) |
| rs718099 | 41,816,354 | *DSCAM* | intronic | A | C | 0.149 | 0.137 |  | 0.3622 | 1.11( 0.89- 1.37) |
| rs62225465 | 41,817,198 | *DSCAM* | intronic | A | C | 0.960 | 0.947 |  | 0.1427 | 1.32( 0.91- 1.93) |
| rs2222973 | 41,833,884 | *DSCAM* | intronic | G | A | 0.308 | 0.287 |  | 0.2326 | 1.11( 0.94- 1.30) |
| rs13046277 | 41,837,293 | *DSCAM* | intronic | G | A | 0.804 | 0.778 |  | 0.1132 | 1.17( 0.96- 1.41) |
| rs2837649 | 41,850,636 | *DSCAM* | intronic | A | G | 0.892 | 0.888 |  | 0.7434 | 1.04( 0.82- 1.33) |
| rs56727890 | 41,878,114 | *DSCAM* | intronic | A | C | 0.113 | 0.089 |  | 0.0339 | 1.3( 1.02- 1.66) |
| rs2837665 | 41,879,326 | *DSCAM* | intronic | G | A | 0.683 | 0.681 |  | 0.8872 | 1.01( 0.86- 1.19) |
| rs2837680 | 41,904,108 | *DSCAM* | intronic | A | G | 0.174 | 0.142 |  | 0.021 | 1.27( 1.04- 1.56) |
| rs2837687 | 41,906,815 | *DSCAM* | intronic | G | A | 0.301 | 0.298 |  | 0.8468 | 1.02( 0.86- 1.20) |
| rs2837691 | 41,909,335 | *DSCAM* | intronic | G | A | 0.155 | 0.136 |  | 0.1569 | 1.17( 0.94- 1.44) |
| rs11908987 | 41,910,647 | *DSCAM* | intronic | G | A | 0.355 | 0.333 |  | 0.2275 | 1.1( 0.94- 1.29) |
| rs78209064 | 41,917,971 | *DSCAM* | intronic | A | G | 0.923 | 0.921 |  | 0.8778 | 1.02( 0.77- 1.36) |
| rs13046433 | 41,918,117 | *DSCAM* | intronic | G | A | 0.101 | 0.091 |  | 0.3434 | 1.13( 0.88- 1.46) |
| rs13050543 | 41,924,389 | *DSCAM* | intronic | G | A | 0.106 | 0.104 |  | 0.8421 | 1.03( 0.80- 1.31) |
| rs56173150 | 41,928,606 | *DSCAM* | intronic | C | A | 0.668 | 0.648 |  | 0.2765 | 1.09( 0.93- 1.28) |
| rs2837706 | 41,932,078 | *DSCAM* | intronic | A | G | 0.713 | 0.676 |  | 0.0403 | 1.19( 1.01- 1.41) |
| rs461728 | 41,956,126 | *DSCAM* | intronic | G | A | 0.738 | 0.697 |  | 0.0187 | 1.23( 1.03- 1.46) |
| rs460699 | 41,964,695 | *DSCAM* | intronic | A | G | 0.491 | 0.446 |  | 0.0197 | 1.2( 1.03- 1.39) |
| rs2837756 | 41,995,874 | *DSCAM* | intronic | G | A | 0.438 | 0.389 |  | 0.0091 | 1.23( 1.05- 1.43) |
| rs73368980 | 42,003,285 | *DSCAM* | intronic | A | G | 0.064 | 0.058 |  | 0.5092 | 1.11( 0.81- 1.52) |
| rs2142125 | 42,010,384 | *DSCAM* | intronic | G | A | 0.178 | 0.163 |  | 0.3121 | 1.11( 0.91- 1.35) |
| rs75849032 | 42,020,025 | *DSCAM* | intronic | G | A | 0.400 | 0.392 |  | 0.6673 | 1.03( 0.89- 1.21) |
| rs200564656 | 42,024,279 | *DSCAM* | intronic | A | C | 0.719 | 0.716 |  | 0.874 | 1.01( 0.86- 1.20) |
| rs2837766 | 42,028,282 | *DSCAM* | intronic | G | A | 0.664 | 0.655 |  | 0.6421 | 1.04( 0.89- 1.22) |
| rs8132096 | 42,030,318 | *DSCAM* | intronic | A | G | 0.495 | 0.483 |  | 0.5221 | 1.05( 0.90- 1.22) |
| rs2837770 | 42,034,352 | *DSCAM* | intronic | G | A | 0.575 | 0.560 |  | 0.4172 | 1.07( 0.91- 1.24) |
| rs75695199 | 42,037,348 | *DSCAM* | intronic | A | G | 0.920 | 0.915 |  | 0.6127 | 1.07( 0.81- 1.42) |
| rs8134673 | 42,048,311 | *DSCAM* | intronic | G | A | 0.581 | 0.567 |  | 0.4549 | 1.06( 0.91- 1.24) |
| rs2837783 | 42,059,090 | *DSCAM* | intronic | A | G | 0.272 | 0.270 |  | 0.896 | 1.01( 0.85- 1.20) |
| rs8132311 | 42,064,088 | *DSCAM* | intronic | A | G | 0.675 | 0.673 |  | 0.924 | 1.01( 0.86- 1.19) |
| rs3804025 | 42,064,507 | *DSCAM* | intronic | G | A | 0.900 | 0.892 |  | 0.5183 | 1.09( 0.85- 1.40) |
| rs12152040 | 42,069,068 | *DSCAM* | intronic | G | A | 0.090 | 0.087 |  | 0.8037 | 1.03( 0.79- 1.35) |
| rs2837793 | 42,087,364 | *DSCAM* | intronic | G | A | 0.767 | 0.748 |  | 0.2531 | 1.11( 0.93- 1.33) |
| rs12626972 | 42,092,530 | *DSCAM* | intronic | A | C | 0.912 | 0.907 |  | 0.6736 | 1.06( 0.81- 1.38) |
| rs7278321 | 42,105,523 | *DSCAM* | intronic | A | G | 0.895 | 0.871 |  | 0.0586 | 1.26( 0.99- 1.61) |
| rs2223006 | 42,113,422 | *DSCAM* | intronic | G | A | 0.668 | 0.658 |  | 0.5884 | 1.05( 0.89- 1.23) |
| rs117139234 | 42,116,943 | *DSCAM* | intronic | A | G | 0.949 | 0.949 |  | 0.9871 | 1( 0.71- 1.41) |
| rs2210270 | 42,132,802 | *DSCAM* | intronic | A | C | 0.437 | 0.414 |  | 0.2305 | 1.1( 0.94- 1.28) |
| rs77603305 | 42,140,696 | *DSCAM* | intronic | G | A | 0.069 | 0.062 |  | 0.4572 | 1.12( 0.83- 1.52) |
| rs12329653 | 42,164,052 | *DSCAM* | intronic | G | A | 0.835 | 0.826 |  | 0.5417 | 1.07( 0.87- 1.31) |
| rs736976 | 42,168,325 | *DSCAM* | intronic | G | A | 0.796 | 0.784 |  | 0.4136 | 1.08( 0.90- 1.30) |
| rs2410291 | 42,169,757 | *DSCAM* | intronic | A | G | 0.918 | 0.911 |  | 0.5425 | 1.09( 0.83- 1.43) |
| rs9305706 | 42,183,125 | *DSCAM* | intronic | A | G | 0.382 | 0.367 |  | 0.4342 | 1.06( 0.91- 1.24) |
| rs7283279 | 42,185,860 | *DSCAM* | intronic | G | A | 0.920 | 0.919 |  | 0.9191 | 1.02( 0.77- 1.34) |
| rs78823543 | 42,190,846 | *DSCAM* | intronic | C | A | 0.962 | 0.943 |  | 0.0305 | 1.52( 1.04- 2.23) |
| rs57292936 | 42,191,539 | *DSCAM* | intronic | G | A | 0.100 | 0.093 |  | 0.5408 | 1.08( 0.84- 1.40) |
| rs2178826 | 42,195,528 | *DSCAM* | intronic | G | A | 0.063 | 0.052 |  | 0.2158 | 1.22( 0.89- 1.68) |
| rs9975158 | 42,198,154 | *DSCAM* | intronic | A | G | 0.695 | 0.688 |  | 0.6972 | 1.03( 0.88- 1.22) |
| rs2898425 | 42,201,615 | *DSCAM* | intronic | A | G | 0.243 | 0.208 |  | 0.0271 | 1.22( 1.02- 1.46) |
| rs932241 | 42,208,057 | *DSCAM* | intronic | A | C | 0.794 | 0.790 |  | 0.7718 | 1.03( 0.85- 1.24) |
| rs73903031 | 42,208,121 | *DSCAM* | intronic | A | G | 0.218 | 0.202 |  | 0.3219 | 1.1( 0.91- 1.32) |
| rs35480596 | 42,210,001 | *DSCAM* | intronic | A | C | 0.550 | 0.546 |  | 0.8468 | 1.02( 0.87- 1.18) |
| rs980274 | 42,212,238 | *DSCAM* | intronic | G | A | 0.698 | 0.694 |  | 0.8275 | 1.02( 0.86- 1.20) |
| rs9981347 | 42,543,537 | *BACE2* | intronic | G | A | 0.769 | 0.761 |  | 0.6493 | 1.04( 0.87- 1.25) |
| rs914177 | 42,546,461 | *BACE2* | intronic | A | G | 0.062 | 0.058 |  | 0.6524 | 1.08( 0.78- 1.48) |
| rs59066201 | 42,548,224 | *BACE2* | intronic | A | C | 0.674 | 0.664 |  | 0.6001 | 1.04( 0.89- 1.23) |
| rs73222244 | 42,558,997 | *BACE2* | intronic | G | A | 0.918 | 0.902 |  | 0.1635 | 1.21( 0.92- 1.59) |
| rs730513 | 42,566,519 | *BACE2* | intronic | G | A | 0.900 | 0.882 |  | 0.1364 | 1.21( 0.94- 1.55) |
| rs730514 | 42,566,585 | *BACE2* | intronic | G | A | 0.873 | 0.857 |  | 0.2365 | 1.15( 0.91- 1.43) |
| rs73222254 | 42,572,514 | *BACE2* | intronic | G | A | 0.942 | 0.928 |  | 0.1529 | 1.26( 0.92- 1.73) |
| rs3787933 | 42,573,589 | *BACE2* | intronic | A | G | 0.937 | 0.932 |  | 0.576 | 1.09( 0.80- 1.49) |
| rs737287 | 42,581,703 | *BACE2* | intronic | G | A | 0.931 | 0.929 |  | 0.8069 | 1.04( 0.77- 1.40) |
| rs113072292 | 42,591,078 | *BACE2* | intronic | G | A | 0.939 | 0.936 |  | 0.7142 | 1.06( 0.77- 1.45) |
| rs28629220 | 42,597,387 | *BACE2* | intronic | A | G | 0.918 | 0.915 |  | 0.7912 | 1.04( 0.79- 1.37) |
| rs7278856 | 42,598,887 | *BACE2* | intronic | A | C | 0.844 | 0.840 |  | 0.7552 | 1.03( 0.84- 1.27) |
| rs35470608 | 42,606,757 | *BACE2* | intronic | A | G | 0.951 | 0.948 |  | 0.7122 | 1.07( 0.75- 1.51) |
| rs2009135 | 42,608,231 | *BACE2* | intronic | G | A | 0.134 | 0.133 |  | 0.9624 | 1.01( 0.80- 1.26) |
| rs73366468 | 42,612,880 | *BACE2* | intronic | A | G | 0.076 | 0.076 |  | 0.9598 | 1.01( 0.76- 1.34) |
| rs2837995 | 42,624,470 | *BACE2* | intronic | A | G | 0.095 | 0.086 |  | 0.3764 | 1.13( 0.87- 1.46) |
| rs2837996 | 42,626,706 | *BACE2* | intronic | G | A | 0.920 | 0.920 |  | 0.9645 | 1.01( 0.76- 1.33) |
| rs8134468 | 42,638,685 | *BACE2* | intronic | G | A | 0.785 | 0.777 |  | 0.6203 | 1.05( 0.87- 1.26) |
| rs12149 | 42,647,821 | *BACE2* | 3'-UTR | A | G | 0.700 | 0.694 |  | 0.7151 | 1.03( 0.87- 1.22) |

RAF: Reference allele Frequency

**Supplementary Table 8.** Function annotation of rs430255.

| **chr** | **pos (hg38)** | **LD** | **LD** | **variant** | **Ref** | **Alt** | **AFR** | **AMR** | **ASN** | **EUR** | **Enhancer** | **DNAse** | **Motifs** | **GENCODE** | **dbSNP** |
| --- | --- | --- | --- | --- | --- | --- | --- | --- | --- | --- | --- | --- | --- | --- | --- |
|  |  | **(r²)** | **(D')** |  |  |  | **freq** | **freq** | **freq** | **freq** | **histone marks** |  | **changed** | **genes** | **func annot** |
| 21 | 40124678 | 1 | 1 | rs430255 | A | G | 0.44 | 0.82 | 0.86 | 0.81 |  |  | GZF1,RXRA | DSCAM | intronic |

Rs430255 was queried on HaploReg v4.1 using data of ASN population with r^2^ >= 0.8

**Supplementary Table 9.** Function annotation of rs2837756.

| **chr** | **pos (hg38)** | **LD** | **LD** | **variant** | **Ref** | **Alt** | **AFR** | **AMR** | **ASN** | **EUR** | **Enhancer** | **DNAse** | **Motifs** | **GENCODE** | **dbSNP** |
| --- | --- | --- | --- | --- | --- | --- | --- | --- | --- | --- | --- | --- | --- | --- | --- |
|  |  | **(r²)** | **(D')** |  |  |  | **freq** | **freq** | **freq** | **freq** | **histone marks** |  | **changed** | **genes** | **func annot** |
| 21 | 40623948 | 1 | 1 | rs2837756 | T | C | 0.31 | 0.65 | 0.41 | 0.77 | ESC, IPSC, BRN |  | AP-1,STAT | DSCAM-IT1 | intronic |
| 21 | 40624758 | 0.96 | 1 | rs138418129 | 13-mer | A | 0.3 | 0.64 | 0.4 | 0.77 | ESC, IPSC |  | Foxp1,RREB-1 | DSCAM-IT1 | intronic |
| 21 | 40625603 | 0.92 | 1 | rs9974484 | C | T | 0.25 | 0.53 | 0.39 | 0.6 | BRN | BLD | Nkx3 | DSCAM-IT1 | intronic |
| 21 | 40627481 | 0.91 | 0.98 | rs760353 | A | G | 0.33 | 0.57 | 0.4 | 0.65 |  |  | AP-1,En-1,Hmbox1,Hoxd8,Pou2f2,SIX5 | DSCAM-IT1 | intronic |
| 21 | 40628562 | 0.92 | 0.99 | rs28526702 | T | C | 0.24 | 0.55 | 0.39 | 0.65 |  |  | Zfp187 | DSCAM-IT1 | intronic |

Rs2837756 was queried on HaploReg v4.1 using data of ASN population with r^2^ >= 0.8

**Supplementary Table 10.** Stratification analysis of 133 tag SNPs in 420 unrelated sporadic HSCR patients (323 S-HSCR, 58 L-HSCR and 39 TCA) and 1,665 controls.

| SNP | GENE | Reference allele | RAF | S-HSCR *vs* control | |  | RAF | L-HSCR *vs* control | |  | RAF | TCA *vs* control | |
| --- | --- | --- | --- | --- | --- | --- | --- | --- | --- | --- | --- | --- | --- |
|  |  |  | S-HSCR | *P* | OR 95%CI |  | L-HSCR | *P* | OR 95%CI |  | TCA | *P* | OR 95%CI |
| rs62235573 | *DSCAM* | A | 0.896 | 0.4935 | 1.1( 0.84- 1.45) |  | 0.897 | 0.7496 | 1.1( 0.60- 2.03) |  | 0.910 | 0.5208 | 1.29( 0.59- 2.83) |
| rs9975444 | *DSCAM* | A | 0.950 | 0.6449 | 1.1( 0.74- 1.61) |  | 0.966 | 0.3547 | 1.6( 0.58- 4.40) |  | 0.987 | 0.1079 | 4.41( 0.61- 31.88) |
| rs9975710 | *DSCAM* | G | 0.088 | 0.6447 | 0.93( 0.69- 1.25) |  | 0.121 | 0.3348 | 1.32( 0.75- 2.34) |  | 0.103 | 0.7978 | 1.1( 0.53- 2.31) |
| rs9981861 | *DSCAM* | A | 0.828 | 0.3131 | 1.12( 0.90- 1.40) |  | 0.819 | 0.8356 | 1.05( 0.65- 1.70) |  | 0.821 | 0.8370 | 1.06( 0.59- 1.91) |
| rs2142123 | *DSCAM* | G | 0.867 | 0.3251 | 1.13( 0.88- 1.45) |  | 0.879 | 0.4135 | 1.27( 0.72- 2.23) |  | 0.833 | 0.6476 | 0.87( 0.48- 1.59) |
| rs2837402 | *DSCAM* | G | 0.071 | 0.8014 | 1.04( 0.75- 1.45) |  | 0.095 | 0.272 | 1.43( 0.75- 2.69) |  | 0.077 | 0.7704 | 1.13( 0.49- 2.64) |
| rs11701316 | *DSCAM* | A | 0.857 | 0.3636 | 1.12( 0.88- 1.42) |  | 0.853 | 0.7608 | 1.09( 0.64- 1.83) |  | 0.842 | 0.9832 | 0.99( 0.53- 1.85) |
| rs7276767 | *DSCAM* | A | 0.122 | 0.1169 | 1.23( 0.95- 1.60) |  | 0.121 | 0.5053 | 1.21( 0.69- 2.15) |  | 0.105 | 0.9173 | 1.04( 0.50- 2.18) |
| rs373996 | *DSCAM* | A | 0.251 | 0.9733 | 1( 0.83- 1.22) |  | 0.310 | 0.1422 | 1.35( 0.90- 2.02) |  | 0.192 | 0.2428 | 0.71( 0.40- 1.26) |
| rs75667921 | *DSCAM* | G | 0.134 | 0.9231 | 1.01( 0.79- 1.30) |  | 0.132 | 0.9863 | 1( 0.57- 1.73) |  | 0.154 | 0.5762 | 1.19( 0.64- 2.23) |
| rs2837426 | *DSCAM* | G | 0.288 | 0.4551 | 1.07( 0.89- 1.29) |  | 0.241 | 0.4439 | 0.84( 0.55- 1.30) |  | 0.308 | 0.5044 | 1.18( 0.73- 1.92) |
| rs1734936 | *DSCAM* | A | 0.373 | 0.9626 | 1( 0.84- 1.20) |  | 0.336 | 0.4366 | 0.86( 0.58- 1.27) |  | 0.423 | 0.3536 | 1.24( 0.79- 1.95) |
| rs2837430 | *DSCAM* | G | 0.646 | 0.3953 | 1.08( 0.91- 1.29) |  | 0.647 | 0.6892 | 1.08( 0.73- 1.59) |  | 0.654 | 0.6444 | 1.12( 0.70- 1.79) |
| rs17828614 | *DSCAM* | G | 0.937 | 0.2259 | 1.23( 0.88- 1.74) |  | 0.940 | 0.5028 | 1.3( 0.60- 2.83) |  | 0.923 | 0.9934 | 1.00( 0.43- 2.33) |
| rs430255 | *DSCAM* | G | 0.864 | 0.0088 | 1.38( 1.08- 1.76) |  | 0.853 | 0.3735 | 1.27( 0.75- 2.14) |  | 0.859 | 0.3900 | 1.33( 0.70- 2.52) |
| rs2837445 | *DSCAM* | G | 0.259 | 0.6656 | 1.04( 0.86- 1.27) |  | 0.276 | 0.5351 | 1.14( 0.75- 1.73) |  | 0.256 | 0.9044 | 1.03( 0.62- 1.73) |
| rs57550834 | *DSCAM* | A | 0.305 | 0.3051 | 1.1( 0.92- 1.32) |  | 0.259 | 0.536 | 0.88( 0.57- 1.34) |  | 0.333 | 0.3503 | 1.25( 0.78- 2.02) |
| rs2837455 | *DSCAM* | G | 0.362 | 0.7090 | 0.97( 0.81- 1.15) |  | 0.457 | 0.057 | 1.43( 0.99- 2.08) |  | 0.346 | 0.6666 | 0.9( 0.56- 1.45) |
| rs75557627 | *DSCAM* | A | 0.881 | 0.7326 | 1.05( 0.81- 1.36) |  | 0.897 | 0.5076 | 1.23( 0.67- 2.25) |  | 0.885 | 0.8189 | 1.09( 0.54- 2.19) |
| rs73225241 | *DSCAM* | G | 0.951 | 0.2374 | 1.26( 0.86- 1.85) |  | 0.948 | 0.664 | 1.2( 0.52- 2.77) |  | 0.936 | 0.9265 | 0.96( 0.38- 2.40) |
| rs73362149 | *DSCAM* | A | 0.201 | 0.4648 | 1.08( 0.88- 1.34) |  | 0.181 | 0.8317 | 0.95( 0.59- 1.54) |  | 0.180 | 0.8338 | 0.94( 0.52- 1.69) |
| rs8132673 | *DSCAM* | C | 0.714 | 0.8061 | 0.98( 0.81- 1.18) |  | 0.776 | 0.1751 | 1.36( 0.87- 2.11) |  | 0.680 | 0.4508 | 0.83( 0.51- 1.35) |
| rs2837464 | *DSCAM* | A | 0.358 | 0.1118 | 1.15( 0.97- 1.38) |  | 0.267 | 0.1879 | 0.76( 0.50- 1.15) |  | 0.397 | 0.1802 | 1.37( 0.86- 2.16) |
| rs7275239 | *DSCAM* | A | 0.738 | 0.4384 | 1.08( 0.89- 1.31) |  | 0.750 | 0.5176 | 1.15( 0.75- 1.77) |  | 0.756 | 0.5102 | 1.19( 0.71- 2.01) |
| rs2837466 | *DSCAM* | G | 0.297 | 0.7400 | 1.03( 0.86- 1.24) |  | 0.319 | 0.5037 | 1.15( 0.77- 1.70) |  | 0.295 | 0.9294 | 1.02( 0.62- 1.67) |
| rs16999420 | *DSCAM* | A | 0.953 | 0.1704 | 1.32( 0.89- 1.95) |  | 0.991 | 0.0211 | 7.26( 1.01- 52.28) |  | 0.949 | 0.7380 | 1.19( 0.43- 3.29) |
| rs7280615 | *DSCAM* | G | 0.276 | 0.4560 | 1.08( 0.89- 1.30) |  | 0.353 | 0.0272 | 1.54( 1.05- 2.28) |  | 0.205 | 0.2627 | 0.73( 0.42- 1.27) |
| rs8130234 | *DSCAM* | G | 0.714 | 0.1542 | 1.14( 0.95- 1.38) |  | 0.741 | 0.2002 | 1.32( 0.86- 2.01) |  | 0.641 | 0.4058 | 0.82( 0.51- 1.31) |
| rs760174 | *DSCAM* | A | 0.372 | 0.0091 | 1.26( 1.06- 1.51) |  | 0.285 | 0.4336 | 0.85( 0.56- 1.28) |  | 0.346 | 0.6101 | 1.13( 0.71- 1.81) |
| rs7278294 | *DSCAM* | A | 0.663 | 0.4078 | 1.08( 0.90- 1.29) |  | 0.612 | 0.4524 | 0.86( 0.59- 1.26) |  | 0.628 | 0.7449 | 0.93( 0.58- 1.47) |
| rs78800938 | *DSCAM* | A | 0.138 | 0.0518 | 1.28( 1.00- 1.64) |  | 0.078 | 0.2529 | 0.67( 0.34- 1.34) |  | 0.077 | 0.3371 | 0.66( 0.29- 1.54) |
| rs8127634 | *DSCAM* | A | 0.302 | 0.9079 | 1.01( 0.84- 1.22) |  | 0.310 | 0.8036 | 1.05( 0.71- 1.57) |  | 0.282 | 0.7382 | 0.92( 0.56- 1.51) |
| rs2837499 | *DSCAM* | G | 0.623 | 0.9797 | 1( 0.84- 1.19) |  | 0.690 | 0.1421 | 1.35( 0.90- 2.01) |  | 0.654 | 0.5725 | 1.15( 0.71- 1.84) |
| rs76045869 | *DSCAM* | G | 0.797 | 0.4872 | 1.08( 0.87- 1.33) |  | 0.845 | 0.1217 | 1.49( 0.90- 2.48) |  | 0.833 | 0.3033 | 1.37( 0.75- 2.50) |
| rs717416 | *DSCAM* | A | 0.638 | 0.9362 | 0.99( 0.83- 1.18) |  | 0.698 | 0.1939 | 1.31( 0.87- 1.95) |  | 0.744 | 0.0579 | 1.64( 0.98- 2.73) |
| rs73227014 | *DSCAM* | C | 0.107 | 0.0344 | 1.35( 1.02- 1.78) |  | 0.103 | 0.3948 | 1.3( 0.71- 2.40) |  | 0.077 | 0.8867 | 0.94( 0.41- 2.18) |
| rs8133535 | *DSCAM* | G | 0.153 | 0.5719 | 1.07( 0.85- 1.35) |  | 0.155 | 0.7521 | 1.09( 0.65- 1.81) |  | 0.103 | 0.2948 | 0.68( 0.32- 1.41) |
| rs2837565 | *DSCAM* | G | 0.395 | 0.0523 | 1.19( 1.00- 1.41) |  | 0.336 | 0.683 | 0.92( 0.62- 1.36) |  | 0.269 | 0.1186 | 0.67( 0.40- 1.11) |
| rs34336407 | *DSCAM* | A | 0.115 | 0.2747 | 1.16( 0.89- 1.52) |  | 0.132 | 0.2765 | 1.36( 0.78- 2.37) |  | 0.090 | 0.7587 | 0.88( 0.40- 1.94) |
| rs2026272 | *DSCAM* | C | 0.370 | 0.9308 | 1.01( 0.85- 1.20) |  | 0.379 | 0.8068 | 1.05( 0.72- 1.54) |  | 0.487 | 0.0315 | 1.63( 1.04- 2.56) |
| rs1571717 | *DSCAM* | G | 0.433 | 0.6843 | 1.04( 0.87- 1.23) |  | 0.466 | 0.3832 | 1.18( 0.81- 1.71) |  | 0.372 | 0.3491 | 0.80( 0.50- 1.28) |
| rs2205081 | *DSCAM* | A | 0.477 | 0.8813 | 1.01( 0.86- 1.20) |  | 0.500 | 0.5753 | 1.11( 0.77- 1.61) |  | 0.449 | 0.6638 | 0.90( 0.58- 1.42) |
| rs2837583 | *DSCAM* | A | 0.234 | 0.5314 | 1.07( 0.87- 1.30) |  | 0.216 | 0.8584 | 0.96( 0.61- 1.51) |  | 0.192 | 0.5255 | 0.83( 0.47- 1.47) |
| rs2210268 | *DSCAM* | A | 0.792 | 0.4526 | 1.08( 0.88- 1.33) |  | 0.767 | 0.7733 | 0.94( 0.60- 1.45) |  | 0.795 | 0.7313 | 1.10( 0.63- 1.92) |
| rs2837611 | *DSCAM* | A | 0.558 | 0.1607 | 1.13( 0.95- 1.34) |  | 0.569 | 0.3772 | 1.18( 0.81- 1.72) |  | 0.590 | 0.2750 | 1.29( 0.82- 2.03) |
| rs76750953 | *DSCAM* | A | 0.783 | 0.7962 | 1.03( 0.84- 1.26) |  | 0.845 | 0.0904 | 1.55( 0.93- 2.58) |  | 0.846 | 0.1549 | 1.56( 0.84- 2.91) |
| rs56342251 | *DSCAM* | A | 0.848 | 0.7591 | 0.96( 0.76- 1.22) |  | 0.871 | 0.5958 | 1.16( 0.67- 2.01) |  | 0.885 | 0.4345 | 1.32( 0.66- 2.66) |
| rs1882755 | *DSCAM* | A | 0.202 | 0.6088 | 1.06( 0.86- 1.31) |  | 0.224 | 0.4068 | 1.21( 0.77- 1.88) |  | 0.154 | 0.3840 | 0.76( 0.41- 1.41) |
| rs1882757 | *DSCAM* | A | 0.387 | 0.9508 | 0.99( 0.84- 1.18) |  | 0.431 | 0.3534 | 1.19( 0.82- 1.74) |  | 0.526 | 0.0140 | 1.75( 1.11- 2.74) |
| rs2837620 | *DSCAM* | G | 0.579 | 0.8639 | 0.99( 0.83- 1.17) |  | 0.612 | 0.5265 | 1.13( 0.77- 1.65) |  | 0.680 | 0.0860 | 1.52( 0.94- 2.46) |
| rs8129283 | *DSCAM* | A | 0.096 | 0.7348 | 1.05( 0.79- 1.40) |  | 0.138 | 0.0928 | 1.58( 0.92- 2.72) |  | 0.158 | 0.0499 | 1.86( 0.99- 3.48) |
| rs10439672 | *DSCAM* | G | 0.559 | 0.4369 | 1.07( 0.90- 1.27) |  | 0.543 | 0.9871 | 1( 0.69- 1.46) |  | 0.526 | 0.7698 | 0.94( 0.60- 1.47) |
| rs718099 | *DSCAM* | A | 0.135 | 0.8942 | 0.98( 0.77- 1.26) |  | 0.190 | 0.1041 | 1.48( 0.92- 2.38) |  | 0.205 | 0.0832 | 1.63( 0.93- 2.85) |
| rs62225465 | *DSCAM* | A | 0.964 | 0.0658 | 1.51( 0.97- 2.35) |  | 0.940 | 0.7235 | 0.87( 0.40- 1.89) |  | 0.949 | 0.9511 | 1.03( 0.37- 2.86) |
| rs2222973 | *DSCAM* | G | 0.305 | 0.3680 | 1.09( 0.91- 1.31) |  | 0.371 | 0.052 | 1.46( 0.99- 2.15) |  | 0.244 | 0.3977 | 0.80( 0.47- 1.35) |
| rs13046277 | *DSCAM* | G | 0.794 | 0.3761 | 1.1( 0.89- 1.35) |  | 0.853 | 0.0547 | 1.66( 0.98- 2.79) |  | 0.808 | 0.5373 | 1.20( 0.68- 2.11) |
| rs2837649 | *DSCAM* | A | 0.893 | 0.6842 | 1.06( 0.81- 1.39) |  | 0.871 | 0.5696 | 0.85( 0.49- 1.48) |  | 0.910 | 0.5318 | 1.28( 0.59- 2.81) |
| rs56727890 | *DSCAM* | A | 0.115 | 0.0425 | 1.32( 1.01- 1.73) |  | 0.121 | 0.2445 | 1.4( 0.79- 2.48) |  | 0.090 | 0.9865 | 1.01( 0.46- 2.21) |
| rs2837665 | *DSCAM* | G | 0.672 | 0.6554 | 0.96( 0.80- 1.15) |  | 0.750 | 0.1152 | 1.41( 0.92- 2.16) |  | 0.680 | 0.9807 | 0.99( 0.61- 1.61) |
| rs2837680 | *DSCAM* | A | 0.173 | 0.0402 | 1.27( 1.01- 1.59) |  | 0.198 | 0.0904 | 1.49( 0.94- 2.38) |  | 0.141 | 0.9780 | 0.99( 0.52- 1.89) |
| rs2837687 | *DSCAM* | G | 0.271 | 0.1697 | 0.88( 0.73- 1.06) |  | 0.431 | 0.0021 | 1.79( 1.23- 2.60) |  | 0.359 | 0.2433 | 1.32( 0.83- 2.11) |
| rs2837691 | *DSCAM* | G | 0.152 | 0.2846 | 1.14( 0.90- 1.44) |  | 0.198 | 0.0551 | 1.57( 0.99- 2.51) |  | 0.115 | 0.6021 | 0.83( 0.41- 1.67) |
| rs11908987 | *DSCAM* | G | 0.331 | 0.9424 | 0.99( 0.83- 1.19) |  | 0.457 | 0.0054 | 1.69( 1.16- 2.45) |  | 0.397 | 0.2311 | 1.32( 0.84- 2.09) |
| rs78209064 | *DSCAM* | A | 0.921 | 0.9978 | 1( 0.73- 1.37) |  | 0.957 | 0.1561 | 1.9( 0.77- 4.71) |  | 0.885 | 0.2409 | 0.66( 0.32- 1.33) |
| rs13046433 | *DSCAM* | G | 0.098 | 0.5722 | 1.09( 0.82- 1.45) |  | 0.121 | 0.2731 | 1.37( 0.78- 2.43) |  | 0.103 | 0.7211 | 1.14( 0.55- 2.40) |
| rs13050543 | *DSCAM* | G | 0.107 | 0.8070 | 1.04( 0.79- 1.36) |  | 0.078 | 0.3642 | 0.73( 0.37- 1.45) |  | 0.141 | 0.2855 | 1.42( 0.74- 2.71) |
| rs56173150 | *DSCAM* | C | 0.676 | 0.1839 | 1.13( 0.94- 1.35) |  | 0.664 | 0.7299 | 1.07( 0.72- 1.59) |  | 0.615 | 0.5484 | 0.87( 0.55- 1.38) |
| rs2837706 | *DSCAM* | A | 0.711 | 0.0872 | 1.18( 0.98- 1.41) |  | 0.733 | 0.2005 | 1.31( 0.86- 1.99) |  | 0.705 | 0.5902 | 1.15( 0.70- 1.87) |
| rs461728 | *DSCAM* | G | 0.746 | 0.0117 | 1.28( 1.06- 1.55) |  | 0.733 | 0.4057 | 1.19( 0.79- 1.81) |  | 0.680 | 0.7439 | 0.92( 0.57- 1.49) |
| rs460699 | *DSCAM* | A | 0.509 | 0.0030 | 1.29( 1.09- 1.53) |  | 0.457 | 0.8106 | 1.05( 0.72- 1.52) |  | 0.385 | 0.2836 | 0.78( 0.49- 1.23) |
| rs2837756 | *DSCAM* | G | 0.454 | 0.0021 | 1.31( 1.10- 1.55) |  | 0.388 | 0.9857 | 1( 0.68- 1.46) |  | 0.385 | 0.9409 | 0.98( 0.62- 1.56) |
| rs73368980 | *DSCAM* | A | 0.059 | 0.9553 | 1.01( 0.71- 1.45) |  | 0.095 | 0.1017 | 1.69( 0.89- 3.21) |  | 0.064 | 0.8277 | 1.11( 0.44- 2.77) |
| rs2142125 | *DSCAM* | G | 0.188 | 0.1253 | 1.19( 0.95- 1.48) |  | 0.172 | 0.7933 | 1.07( 0.65- 1.74) |  | 0.103 | 0.1503 | 0.59( 0.28- 1.22) |
| rs75849032 | *DSCAM* | G | 0.395 | 0.8922 | 1.01( 0.85- 1.20) |  | 0.414 | 0.6349 | 1.1( 0.75- 1.60) |  | 0.423 | 0.5772 | 1.14( 0.72- 1.79) |
| rs200564656 | *DSCAM* | A | 0.711 | 0.7987 | 0.98( 0.81- 1.18) |  | 0.754 | 0.3725 | 1.22( 0.79- 1.88) |  | 0.731 | 0.7768 | 1.08( 0.65- 1.78) |
| rs2837766 | *DSCAM* | G | 0.655 | 0.9873 | 1( 0.84- 1.20) |  | 0.690 | 0.4393 | 1.17( 0.78- 1.75) |  | 0.692 | 0.4925 | 1.19( 0.73- 1.93) |
| rs8132096 | *DSCAM* | A | 0.506 | 0.2777 | 1.1( 0.93- 1.30) |  | 0.456 | 0.5745 | 0.9( 0.62- 1.31) |  | 0.462 | 0.7095 | 0.92( 0.59- 1.44) |
| rs2837770 | *DSCAM* | G | 0.579 | 0.3608 | 1.08( 0.91- 1.28) |  | 0.543 | 0.7273 | 0.94( 0.65- 1.36) |  | 0.590 | 0.5943 | 1.13( 0.72- 1.79) |
| rs75695199 | *DSCAM* | A | 0.922 | 0.5168 | 1.11( 0.81- 1.52) |  | 0.914 | 0.9752 | 0.99( 0.51- 1.91) |  | 0.910 | 0.8918 | 0.95( 0.43- 2.08) |
| rs8134673 | *DSCAM* | G | 0.587 | 0.3469 | 1.09( 0.91- 1.29) |  | 0.535 | 0.4918 | 0.88( 0.61- 1.27) |  | 0.603 | 0.5270 | 1.16( 0.73- 1.83) |
| rs2837783 | *DSCAM* | A | 0.290 | 0.2842 | 1.11( 0.92- 1.33) |  | 0.241 | 0.4969 | 0.86( 0.56- 1.33) |  | 0.167 | 0.0419 | 0.54( 0.30- 0.99) |
| rs8132311 | *DSCAM* | A | 0.700 | 0.1886 | 1.13( 0.94- 1.36) |  | 0.647 | 0.5467 | 0.89( 0.60- 1.31) |  | 0.513 | 0.0029 | 0.51( 0.33- 0.80) |
| rs3804025 | *DSCAM* | G | 0.905 | 0.3182 | 1.16( 0.87- 1.54) |  | 0.914 | 0.4571 | 1.28( 0.66- 2.48) |  | 0.833 | 0.1003 | 0.61( 0.33- 1.11) |
| rs12152040 | *DSCAM* | G | 0.081 | 0.6163 | 0.92( 0.68- 1.26) |  | 0.086 | 0.9826 | 0.99( 0.51- 1.92) |  | 0.167 | 0.0141 | 2.10( 1.15- 3.86) |
| rs2837793 | *DSCAM* | G | 0.785 | 0.0445 | 1.23( 1.01- 1.51) |  | 0.724 | 0.5679 | 0.89( 0.59- 1.34) |  | 0.680 | 0.1719 | 0.72( 0.44- 1.16) |
| rs12626972 | *DSCAM* | A | 0.920 | 0.3195 | 1.17( 0.86- 1.59) |  | 0.888 | 0.4832 | 0.81( 0.45- 1.46) |  | 0.885 | 0.4977 | 0.78( 0.39- 1.59) |
| rs7278321 | *DSCAM* | A | 0.898 | 0.0603 | 1.3( 0.99- 1.71) |  | 0.871 | 0.9879 | 1( 0.57- 1.73) |  | 0.910 | 0.3070 | 1.50( 0.69- 3.28) |
| rs2223006 | *DSCAM* | G | 0.669 | 0.5969 | 1.05( 0.88- 1.26) |  | 0.672 | 0.7469 | 1.07( 0.72- 1.58) |  | 0.654 | 0.9397 | 0.98( 0.61- 1.57) |
| rs117139234 | *DSCAM* | A | 0.946 | 0.7649 | 0.94( 0.65- 1.37) |  | 0.948 | 0.9849 | 0.99( 0.43- 2.29) |  | 0.974 | 0.3069 | 2.06( 0.50- 8.44) |
| rs2210270 | *DSCAM* | A | 0.437 | 0.2893 | 1.1( 0.92- 1.30) |  | 0.431 | 0.7153 | 1.07( 0.74- 1.56) |  | 0.449 | 0.5392 | 1.15( 0.73- 1.81) |
| rs77603305 | *DSCAM* | G | 0.061 | 0.8776 | 0.97( 0.68- 1.39) |  | 0.078 | 0.5063 | 1.27( 0.63- 2.53) |  | 0.128 | 0.0187 | 2.21( 1.12- 4.36) |
| rs12329653 | *DSCAM* | G | 0.839 | 0.4566 | 1.09( 0.87- 1.37) |  | 0.836 | 0.7844 | 1.07( 0.65- 1.77) |  | 0.808 | 0.6662 | 0.88( 0.50- 1.56) |
| rs736976 | *DSCAM* | G | 0.805 | 0.2226 | 1.14( 0.92- 1.41) |  | 0.785 | 0.9795 | 1.01( 0.64- 1.58) |  | 0.744 | 0.3985 | 0.8( 0.48- 1.34) |
| rs2410291 | *DSCAM* | A | 0.924 | 0.2871 | 1.19( 0.87- 1.63) |  | 0.905 | 0.8285 | 0.93( 0.50- 1.76) |  | 0.885 | 0.4199 | 0.75( 0.37- 1.52) |
| rs9305706 | *DSCAM* | A | 0.389 | 0.3055 | 1.1( 0.92- 1.30) |  | 0.395 | 0.5499 | 1.12( 0.77- 1.65) |  | 0.308 | 0.2803 | 0.77( 0.47- 1.25) |
| rs7283279 | *DSCAM* | G | 0.924 | 0.6696 | 1.07( 0.78- 1.47) |  | 0.922 | 0.8997 | 1.05( 0.52- 2.09) |  | 0.885 | 0.2705 | 0.67( 0.33- 1.37) |
| rs78823543 | *DSCAM* | C | 0.963 | 0.0422 | 1.56( 1.01- 2.41) |  | 0.957 | 0.5287 | 1.34( 0.54- 3.32) |  | 0.962 | 0.4871 | 1.51( 0.47- 4.82) |
| rs57292936 | *DSCAM* | G | 0.099 | 0.6338 | 1.07( 0.81- 1.42) |  | 0.112 | 0.4906 | 1.23( 0.68- 2.22) |  | 0.090 | 0.9198 | 0.96( 0.44- 2.11) |
| rs2178826 | *DSCAM* | G | 0.065 | 0.1903 | 1.26( 0.89- 1.79) |  | 0.060 | 0.7009 | 1.17( 0.53- 2.54) |  | 0.051 | 0.9696 | 0.98( 0.35- 2.71) |
| rs9975158 | *DSCAM* | A | 0.689 | 0.9773 | 1( 0.84- 1.20) |  | 0.716 | 0.5334 | 1.14( 0.76- 1.72) |  | 0.718 | 0.5759 | 1.15( 0.70- 1.90) |
| rs2898425 | *DSCAM* | A | 0.249 | 0.0189 | 1.27( 1.04- 1.54) |  | 0.241 | 0.382 | 1.21( 0.79- 1.87) |  | 0.192 | 0.7386 | 0.91( 0.51- 1.60) |
| rs932241 | *DSCAM* | A | 0.788 | 0.9289 | 0.99( 0.81- 1.22) |  | 0.828 | 0.3214 | 1.28( 0.78- 2.09) |  | 0.795 | 0.9082 | 1.03( 0.59- 1.80) |
| rs73903031 | *DSCAM* | A | 0.218 | 0.3607 | 1.1( 0.90- 1.35) |  | 0.190 | 0.7367 | 0.92( 0.58- 1.48) |  | 0.256 | 0.2417 | 1.36( 0.81- 2.28) |
| rs35480596 | *DSCAM* | A | 0.536 | 0.6524 | 0.96( 0.81- 1.14) |  | 0.634 | 0.066 | 1.44( 0.97- 2.13) |  | 0.539 | 0.8947 | 0.97( 0.62- 1.52) |
| rs980274 | *DSCAM* | G | 0.683 | 0.5768 | 0.95( 0.79- 1.14) |  | 0.759 | 0.1354 | 1.39( 0.90- 2.14) |  | 0.731 | 0.4828 | 1.20( 0.72- 1.99) |
| rs9981347 | *BACE2* | G | 0.764 | 0.8718 | 1.02( 0.83- 1.24) |  | 0.793 | 0.425 | 1.2( 0.76- 1.90) |  | 0.769 | 0.8664 | 1.05( 0.61- 1.78) |
| rs914177 | *BACE2* | A | 0.059 | 0.9171 | 1.02( 0.71- 1.46) |  | 0.043 | 0.4992 | 0.73( 0.30- 1.82) |  | 0.115 | 0.0337 | 2.12( 1.04- 4.31) |
| rs59066201 | *BACE2* | A | 0.683 | 0.3638 | 1.09( 0.91- 1.30) |  | 0.595 | 0.1202 | 0.74( 0.51- 1.08) |  | 0.718 | 0.3205 | 1.29( 0.78- 2.12) |
| rs73222244 | *BACE2* | G | 0.920 | 0.1676 | 1.24( 0.91- 1.68) |  | 0.871 | 0.2654 | 0.73( 0.42- 1.27) |  | 0.974 | 0.0325 | 4.12( 1.01- 16.87) |
| rs730513 | *BACE2* | G | 0.896 | 0.2887 | 1.16( 0.88- 1.53) |  | 0.871 | 0.719 | 0.9( 0.52- 1.57) |  | 0.974 | 0.0116 | 5.1( 1.25- 20.84) |
| rs730514 | *BACE2* | G | 0.870 | 0.3773 | 1.12( 0.87- 1.44) |  | 0.845 | 0.7188 | 0.91( 0.55- 1.52) |  | 0.936 | 0.0474 | 2.44( 0.98- 6.07) |
| rs73222254 | *BACE2* | G | 0.946 | 0.0959 | 1.36( 0.95- 1.96) |  | 0.914 | 0.573 | 0.83( 0.43- 1.60) |  | 0.949 | 0.4760 | 1.44( 0.52- 3.98) |
| rs3787933 | *BACE2* | A | 0.940 | 0.4496 | 1.15( 0.81- 1.63) |  | 0.922 | 0.7042 | 0.87( 0.44- 1.75) |  | 0.936 | 0.8789 | 1.07( 0.43- 2.68) |
| rs737287 | *BACE2* | G | 0.932 | 0.7608 | 1.05( 0.75- 1.47) |  | 0.922 | 0.8018 | 0.92( 0.46- 1.83) |  | 0.936 | 0.8026 | 1.12( 0.45- 2.81) |
| rs113072292 | *BACE2* | G | 0.940 | 0.7178 | 1.07( 0.75- 1.52) |  | 0.922 | 0.5633 | 0.82( 0.41- 1.63) |  | 0.962 | 0.3578 | 1.71( 0.54- 5.48) |
| rs28629220 | *BACE2* | A | 0.916 | 0.9073 | 1.02( 0.75- 1.38) |  | 0.905 | 0.7091 | 0.89( 0.47- 1.67) |  | 0.949 | 0.2894 | 1.72( 0.62- 4.73) |
| rs7278856 | *BACE2* | A | 0.850 | 0.5159 | 1.08( 0.85- 1.37) |  | 0.810 | 0.3991 | 0.82( 0.51- 1.31) |  | 0.846 | 0.8768 | 1.05( 0.56- 1.96) |
| rs35470608 | *BACE2* | A | 0.954 | 0.5602 | 1.13( 0.76- 1.67) |  | 0.948 | 0.9913 | 1.01( 0.44- 2.32) |  | 0.936 | 0.6335 | 0.80( 0.32- 2.01) |
| rs2009135 | *BACE2* | G | 0.134 | 0.9723 | 1( 0.78- 1.29) |  | 0.138 | 0.8787 | 1.04( 0.61- 1.78) |  | 0.128 | 0.9012 | 0.96( 0.49- 1.88) |
| rs73366468 | *BACE2* | A | 0.079 | 0.7743 | 1.05( 0.77- 1.43) |  | 0.078 | 0.9391 | 1.03( 0.51- 2.05) |  | 0.051 | 0.4191 | 0.66( 0.24- 1.82) |
| rs2837995 | *BACE2* | A | 0.101 | 0.2171 | 1.2( 0.90- 1.59) |  | 0.078 | 0.7618 | 0.9( 0.45- 1.79) |  | 0.077 | 0.7867 | 0.89( 0.38- 2.07) |
| rs2837996 | *BACE2* | G | 0.920 | 0.9818 | 1( 0.73- 1.36) |  | 0.931 | 0.66 | 1.18( 0.57- 2.44) |  | 0.910 | 0.7600 | 0.88( 0.40- 1.94) |
| rs8134468 | *BACE2* | G | 0.794 | 0.3251 | 1.11( 0.90- 1.37) |  | 0.750 | 0.5 | 0.86( 0.56- 1.32) |  | 0.756 | 0.6728 | 0.89( 0.53- 1.51) |
| rs12149 | *BACE2* | A | 0.711 | 0.3894 | 1.09( 0.90- 1.31) |  | 0.664 | 0.4954 | 0.87( 0.59- 1.29) |  | 0.667 | 0.6114 | 0.88( 0.55- 1.42) |

RAF: Reference allele Frequency

**Supplementary Table 11.** Results of protein-protein interaction analysis conducted on GeneMANIA­.

| Gene 1 | Gene 2 | Weight | Network group | Network |
| --- | --- | --- | --- | --- |
| EDNRB | GFRA1 | 0.013972941 | Co-expression | Wang-Maris-2006 |
| SOX10 | GFRA1 | 0.018100848 | Co-expression | Wang-Maris-2006 |
| SOX10 | EDNRB | 0.018595874 | Co-expression | Wang-Maris-2006 |
| DOK5 | POU3F2 | 0.011665206 | Co-expression | Wang-Maris-2006 |
| EDN2 | RET | 0.010794416 | Co-expression | Wang-Maris-2006 |
| ERBB4 | DOK5 | 0.016296206 | Co-expression | Wang-Maris-2006 |
| NRTN | SEMA3D | 0.017000329 | Co-expression | Rieger-Chu-2004 |
| PHOX2B | NRTN | 0.00941987 | Co-expression | Rieger-Chu-2004 |
| NRG1 | NRTN | 0.015240706 | Co-expression | Rieger-Chu-2004 |
| GFRA2 | SEMA3D | 0.024948163 | Co-expression | Mallon-McKay-2013 |
| SOX10 | DSCAM | 0.006972376 | Co-expression | Mallon-McKay-2013 |
| SOX10 | GFRA1 | 0.004377506 | Co-expression | Mallon-McKay-2013 |
| DOK4 | DOK6 | 0.005275386 | Co-expression | Mallon-McKay-2013 |
| PAX3 | POU3F2 | 0.029612033 | Co-expression | Mallon-McKay-2013 |
| EDN3 | GDNF | 0.009430535 | Co-expression | Dobbin-Giordano-2005 |
| EDN3 | NRTN | 0.008125412 | Co-expression | Dobbin-Giordano-2005 |
| SOX10 | GFRA1 | 0.012862844 | Co-expression | Dobbin-Giordano-2005 |
| ERBB4 | DOK5 | 0.007813661 | Co-expression | Dobbin-Giordano-2005 |
| DOK4 | EDN3 | 0.008498606 | Co-expression | Dobbin-Giordano-2005 |
| GFRA4 | POU3F2 | 0.001825176 | Co-expression | Dobbin-Giordano-2005 |
| PHOX2B | SEMA3D | 0.010459841 | Co-expression | Bild-Nevins-2006 B |
| SOX10 | NRTN | 0.008260364 | Co-expression | Bild-Nevins-2006 B |
| SOX10 | EDN3 | 0.008448018 | Co-expression | Bild-Nevins-2006 B |
| CALCA | DSCAM | 0.013792845 | Co-expression | Bild-Nevins-2006 B |
| KEL | EDN3 | 0.011257857 | Co-expression | Bild-Nevins-2006 B |
| KEL | NRP2 | 0.014240094 | Co-expression | Bild-Nevins-2006 B |
| KEL | CTSE | 0.01725496 | Co-expression | Bild-Nevins-2006 B |
| DSCAM | NRTN | 0.02179019 | Co-expression | Smirnov-Cheung-2009 |
| EDN1 | EDNRB | 0.014084284 | Co-expression | Smirnov-Cheung-2009 |
| DOK5 | SEMA3C | 0.004095503 | Co-expression | Smirnov-Cheung-2009 |
| DOK5 | GFRA1 | 0.003127624 | Co-expression | Smirnov-Cheung-2009 |
| DOK5 | NRG1 | 0.003756829 | Co-expression | Smirnov-Cheung-2009 |
| DOK5 | RET | 0.004705983 | Co-expression | Smirnov-Cheung-2009 |
| ERBB3 | GFRA1 | 0.033017904 | Co-expression | Smirnov-Cheung-2009 |
| ERBB3 | DOK5 | 0.003530109 | Co-expression | Smirnov-Cheung-2009 |
| ERBB4 | DOK5 | 0.004281912 | Co-expression | Smirnov-Cheung-2009 |
| NRP2 | DOK5 | 0.003135641 | Co-expression | Smirnov-Cheung-2009 |
| CTSE | RET | 0.01116817 | Co-expression | Smirnov-Cheung-2009 |
| CTSE | DOK5 | 0.001344392 | Co-expression | Smirnov-Cheung-2009 |
| EDNRB | EDN3 | 0.010395672 | Co-expression | Perou-Botstein-2000 |
| NRG1 | BACE2 | 0.014710555 | Co-expression | Perou-Botstein-2000 |
| EDN1 | GFRA1 | 0.019342665 | Co-expression | Perou-Botstein-2000 |
| GNA11 | ECE1 | 0.017279195 | Co-expression | Perou-Botstein-2000 |
| NRP2 | BACE2 | 0.008442614 | Co-expression | Perou-Botstein-2000 |
| NRP2 | ECE1 | 0.007339622 | Co-expression | Perou-Botstein-2000 |
| CTSE | NRP2 | 0.00729487 | Co-expression | Perou-Botstein-2000 |
| DSCAM | NRTN | 0.005028261 | Co-expression | Wu-Garvey-2007 |
| NRG1 | NRTN | 0.011319264 | Co-expression | Wu-Garvey-2007 |
| CALCA | DSCAM | 0.013421478 | Co-expression | Wu-Garvey-2007 |
| KEL | DSCAM | 0.00894127 | Co-expression | Wu-Garvey-2007 |
| RET | NRTN | 0.010773454 | Co-expression | Wang-Cheung-2015 |
| EDN2 | CALCA | 0.022131706 | Co-expression | Wang-Cheung-2015 |
| GFRA4 | CALCA | 0.024573063 | Co-expression | Wang-Cheung-2015 |
| RET | EDNRB | 0.00822138 | Co-expression | Chen-Brown-2002 |
| RET | GFRA1 | 0.017244315 | Co-expression | Roth-Zlotnik-2006 |
| ERBB3 | SOX10 | 0.020424563 | Co-expression | Roth-Zlotnik-2006 |
| CALCA | RET | 0.015419691 | Co-expression | Roth-Zlotnik-2006 |
| ERBB4 | DSCAM | 0.018247379 | Co-expression | Roth-Zlotnik-2006 |
| EDNRA | SEMA3C | 0.015997816 | Co-expression | Roth-Zlotnik-2006 |
| GFRA1 | DSCAM | 0.006582376 | Co-expression | Noble-Diehl-2008 |
| PAX3 | DSCAM | 0.01128535 | Co-expression | Noble-Diehl-2008 |
| DNAJC5 | GNA11 | 0.006079645 | Co-expression | Noble-Diehl-2008 |
| DNAJC5 | DOK4 | 0.012715258 | Co-expression | Noble-Diehl-2008 |
| GFRA4 | GDNF | 0.013530183 | Co-expression | Noble-Diehl-2008 |
| PHOX2B | NRTN | 0.011624465 | Co-localization | Johnson-Shoemaker-2003 |
| NRG1 | SEMA3C | 0.020016182 | Co-localization | Johnson-Shoemaker-2003 |
| ERBB3 | BACE2 | 0.010301061 | Co-localization | Johnson-Shoemaker-2003 |
| PAX3 | BACE2 | 0.009788138 | Co-localization | Johnson-Shoemaker-2003 |
| GDNF | SEMA3D | 0.000680411 | Genetic Interactions | Lin-Smith-2010 |
| SEMA3C | DSCAM | 0.000564308 | Genetic Interactions | Lin-Smith-2010 |
| PHOX2B | GDNF | 0.00075516 | Genetic Interactions | Lin-Smith-2010 |
| GFRA1 | SEMA3D | 0.000259327 | Genetic Interactions | Lin-Smith-2010 |
| GFRA1 | DSCAM | 0.000304711 | Genetic Interactions | Lin-Smith-2010 |
| GFRA1 | SEMA3C | 0.000301462 | Genetic Interactions | Lin-Smith-2010 |
| GFRA1 | PHOX2B | 0.000287816 | Genetic Interactions | Lin-Smith-2010 |
| EDN3 | SEMA3D | 0.000641794 | Genetic Interactions | Lin-Smith-2010 |
| EDN3 | SEMA3C | 0.000746072 | Genetic Interactions | Lin-Smith-2010 |
| EDN3 | GFRA1 | 0.000402859 | Genetic Interactions | Lin-Smith-2010 |
| EDNRB | DSCAM | 0.000447057 | Genetic Interactions | Lin-Smith-2010 |
| EDNRB | GFRA1 | 0.000238824 | Genetic Interactions | Lin-Smith-2010 |
| BACE2 | SEMA3C | 0.000559917 | Genetic Interactions | Lin-Smith-2010 |
| BACE2 | GFRA1 | 0.00030234 | Genetic Interactions | Lin-Smith-2010 |
| BACE2 | EDN3 | 0.000748245 | Genetic Interactions | Lin-Smith-2010 |
| BACE2 | EDNRB | 0.000443578 | Genetic Interactions | Lin-Smith-2010 |
| NRG1 | GDNF | 0.000472528 | Genetic Interactions | Lin-Smith-2010 |
| NRG1 | NRTN | 0.001161372 | Genetic Interactions | Lin-Smith-2010 |
| NRG1 | SEMA3C | 0.000333527 | Genetic Interactions | Lin-Smith-2010 |
| NRG1 | ECE1 | 0.000320053 | Genetic Interactions | Lin-Smith-2010 |
| SOX10 | GFRA1 | 0.000949578 | Genetic Interactions | Lin-Smith-2010 |
| RET | PHOX2B | 0.000399412 | Genetic Interactions | Lin-Smith-2010 |
| RET | GFRA1 | 0.000225897 | Genetic Interactions | Lin-Smith-2010 |
| RET | EDN3 | 0.000559061 | Genetic Interactions | Lin-Smith-2010 |
| RET | EDNRB | 0.000331425 | Genetic Interactions | Lin-Smith-2010 |
| RET | ECE1 | 0.000401448 | Genetic Interactions | Lin-Smith-2010 |
| DOK6 | GFRA1 | 0.000375291 | Genetic Interactions | Lin-Smith-2010 |
| POU3F2 | EDNRB | 0.000642519 | Genetic Interactions | Lin-Smith-2010 |
| POU3F2 | SOX10 | 0.002554689 | Genetic Interactions | Lin-Smith-2010 |
| DOK5 | GDNF | 0.001067987 | Genetic Interactions | Lin-Smith-2010 |
| DOK5 | GFRA1 | 0.000407045 | Genetic Interactions | Lin-Smith-2010 |
| DOK5 | POU3F2 | 0.001095089 | Genetic Interactions | Lin-Smith-2010 |
| GGA2 | GDNF | 0.000816249 | Genetic Interactions | Lin-Smith-2010 |
| GGA2 | GFRA1 | 0.000311099 | Genetic Interactions | Lin-Smith-2010 |
| GGA2 | EDNRB | 0.000456428 | Genetic Interactions | Lin-Smith-2010 |
| GGA2 | RET | 0.000431723 | Genetic Interactions | Lin-Smith-2010 |
| EDN2 | BACE2 | 0.000582406 | Genetic Interactions | Lin-Smith-2010 |
| EDN2 | GGA2 | 0.000599278 | Genetic Interactions | Lin-Smith-2010 |
| ERBB4 | PHOX2B | 0.000301522 | Genetic Interactions | Lin-Smith-2010 |
| ERBB4 | GFRA1 | 0.000170533 | Genetic Interactions | Lin-Smith-2010 |
| ERBB4 | EDNRB | 0.000250197 | Genetic Interactions | Lin-Smith-2010 |
| ERBB4 | BACE2 | 0.000316738 | Genetic Interactions | Lin-Smith-2010 |
| ERBB4 | NRG1 | 0.000188672 | Genetic Interactions | Lin-Smith-2010 |
| ERBB4 | GGA2 | 0.000325913 | Genetic Interactions | Lin-Smith-2010 |
| ERBB4 | EDN2 | 0.000328502 | Genetic Interactions | Lin-Smith-2010 |
| GNA11 | ERBB4 | 0.00187113 | Genetic Interactions | Lin-Smith-2010 |
| NRP2 | SEMA3C | 0.000336739 | Genetic Interactions | Lin-Smith-2010 |
| NRP2 | GFRA1 | 0.00018183 | Genetic Interactions | Lin-Smith-2010 |
| NRP2 | EDN3 | 0.000450001 | Genetic Interactions | Lin-Smith-2010 |
| NRP2 | EDNRB | 0.000266772 | Genetic Interactions | Lin-Smith-2010 |
| NRP2 | BACE2 | 0.00033772 | Genetic Interactions | Lin-Smith-2010 |
| NRP2 | NRG1 | 0.000201171 | Genetic Interactions | Lin-Smith-2010 |
| NRP2 | DOK6 | 0.000419207 | Genetic Interactions | Lin-Smith-2010 |
| NRP2 | POU3F2 | 0.000489185 | Genetic Interactions | Lin-Smith-2010 |
| NRP2 | DOK5 | 0.000454677 | Genetic Interactions | Lin-Smith-2010 |
| NRP2 | ERBB3 | 0.001558898 | Genetic Interactions | Lin-Smith-2010 |
| NRP2 | GGA2 | 0.000347504 | Genetic Interactions | Lin-Smith-2010 |
| GGA1 | PHOX2B | 0.00185333 | Genetic Interactions | Lin-Smith-2010 |
| PAX3 | PHOX2B | 0.000509115 | Genetic Interactions | Lin-Smith-2010 |
| PAX3 | GFRA1 | 0.000287942 | Genetic Interactions | Lin-Smith-2010 |
| PAX3 | EDNRB | 0.000422454 | Genetic Interactions | Lin-Smith-2010 |
| PAX3 | BACE2 | 0.000534806 | Genetic Interactions | Lin-Smith-2010 |
| PAX3 | ECE1 | 0.000511709 | Genetic Interactions | Lin-Smith-2010 |
| DNAJC5 | ERBB4 | 0.001383021 | Genetic Interactions | Lin-Smith-2010 |
| CTSE | EDNRB | 0.00082973 | Genetic Interactions | Lin-Smith-2010 |
| CTSE | DOK6 | 0.001303846 | Genetic Interactions | Lin-Smith-2010 |
| CTSE | NRP2 | 0.000631719 | Genetic Interactions | Lin-Smith-2010 |
| CTSE | PAX3 | 0.001000377 | Genetic Interactions | Lin-Smith-2010 |
| KEL | EDN3 | 0.002956971 | Genetic Interactions | Lin-Smith-2010 |
| EDNRA | EDNRB | 0.000573101 | Genetic Interactions | Lin-Smith-2010 |
| GFRA4 | NRTN | 0.004801654 | Genetic Interactions | Lin-Smith-2010 |
| GFRA1 | GDNF | 0.024436418 | Pathway | Wu-Stein-2010 |
| EDNRB | EDN3 | 0.13514973 | Pathway | Wu-Stein-2010 |
| ECE1 | EDN3 | 0.27759084 | Pathway | Wu-Stein-2010 |
| RET | GDNF | 0.022846786 | Pathway | Wu-Stein-2010 |
| RET | GFRA1 | 0.029607415 | Pathway | Wu-Stein-2010 |
| DOK6 | GDNF | 0.13123737 | Pathway | Wu-Stein-2010 |
| DOK6 | GFRA1 | 0.17007202 | Pathway | Wu-Stein-2010 |
| DOK6 | RET | 0.15900853 | Pathway | Wu-Stein-2010 |
| EDN1 | EDNRB | 0.027404644 | Pathway | Wu-Stein-2010 |
| EDN1 | ECE1 | 0.056287777 | Pathway | Wu-Stein-2010 |
| DOK5 | GDNF | 0.150393 | Pathway | Wu-Stein-2010 |
| DOK5 | GFRA1 | 0.19489601 | Pathway | Wu-Stein-2010 |
| DOK5 | RET | 0.1822177 | Pathway | Wu-Stein-2010 |
| ERBB3 | NRG1 | 0.047480226 | Pathway | Wu-Stein-2010 |
| EDN2 | EDNRB | 0.13514973 | Pathway | Wu-Stein-2010 |
| EDN2 | ECE1 | 0.27759084 | Pathway | Wu-Stein-2010 |
| GNA11 | EDNRB | 0.015508096 | Pathway | Wu-Stein-2010 |
| GNA11 | EDN1 | 0.005116506 | Pathway | Wu-Stein-2010 |
| DOK4 | GDNF | 0.12607777 | Pathway | Wu-Stein-2010 |
| DOK4 | GFRA1 | 0.16338564 | Pathway | Wu-Stein-2010 |
| DOK4 | RET | 0.15275712 | Pathway | Wu-Stein-2010 |
| EDNRA | EDN3 | 0.09628944 | Pathway | Wu-Stein-2010 |
| EDNRA | EDN1 | 0.019524848 | Pathway | Wu-Stein-2010 |
| EDNRA | EDN2 | 0.09628944 | Pathway | Wu-Stein-2010 |
| EDNRA | GNA11 | 0.011048975 | Pathway | Wu-Stein-2010 |
| GDNF | GFRA2 | 0.46176168 | Physical Interactions | IREF-BIOGRID |
| NRTN | GFRA2 | 0.48946217 | Physical Interactions | IREF-BIOGRID |
| GFRA1 | GDNF | 0.33328643 | Physical Interactions | IREF-BIOGRID |
| GFRA1 | NRTN | 0.35327983 | Physical Interactions | IREF-BIOGRID |
| EDNRB | EDN3 | 0.7998857 | Physical Interactions | IREF-BIOGRID |
| RET | NRTN | 0.13644987 | Physical Interactions | IREF-BIOGRID |
| RET | GFRA1 | 0.09806985 | Physical Interactions | IREF-BIOGRID |
| DOK6 | RET | 0.17954096 | Physical Interactions | IREF-BIOGRID |
| POU3F2 | SOX10 | 0.27780405 | Physical Interactions | IREF-BIOGRID |
| DOK5 | RET | 0.15192088 | Physical Interactions | IREF-BIOGRID |
| ERBB3 | NRG1 | 0.0855292 | Physical Interactions | IREF-BIOGRID |
| CALCA | ECE1 | 0.1671175 | Physical Interactions | IREF-BIOGRID |
| GGA2 | BACE2 | 0.21538667 | Physical Interactions | IREF-BIOGRID |
| LIMK1 | NRG1 | 0.23405686 | Physical Interactions | IREF-BIOGRID |
| ERBB4 | NRG1 | 0.11961702 | Physical Interactions | IREF-BIOGRID |
| GNA11 | EDNRB | 0.14926244 | Physical Interactions | IREF-BIOGRID |
| DOK4 | RET | 0.11126906 | Physical Interactions | IREF-BIOGRID |
| GGA1 | BACE2 | 0.16885902 | Physical Interactions | IREF-BIOGRID |
| GGA1 | GGA2 | 0.021324122 | Physical Interactions | IREF-BIOGRID |
| PAX3 | SOX10 | 0.15110663 | Physical Interactions | IREF-BIOGRID |
| PAX3 | POU3F2 | 0.119657025 | Physical Interactions | IREF-BIOGRID |
| DNAJC5 | ECE1 | 0.19781479 | Physical Interactions | IREF-BIOGRID |
| EDNRA | EDN1 | 0.46039045 | Physical Interactions | IREF-BIOGRID |
| EDNRA | GNA11 | 0.13927712 | Physical Interactions | IREF-BIOGRID |
| GFRA1 | GDNF | 0.70710677 | Physical Interactions | IREF-DIP |
| RET | GFRA1 | 0.70710677 | Physical Interactions | IREF-DIP |
| ERBB3 | NRG1 | 0.5114404 | Physical Interactions | IREF-DIP |
| ERBB4 | NRG1 | 0.30671763 | Physical Interactions | IREF-DIP |
| GDNF | GFRA2 | 0.511004 | Physical Interactions | BIOGRID-SMALL-SCALE-STUDIES |
| NRTN | GFRA2 | 0.4249658 | Physical Interactions | BIOGRID-SMALL-SCALE-STUDIES |
| GFRA1 | GDNF | 0.4866332 | Physical Interactions | BIOGRID-SMALL-SCALE-STUDIES |
| GFRA1 | NRTN | 0.40469837 | Physical Interactions | BIOGRID-SMALL-SCALE-STUDIES |
| EDNRB | EDN3 | 0.7710644 | Physical Interactions | BIOGRID-SMALL-SCALE-STUDIES |
| RET | NRTN | 0.11607487 | Physical Interactions | BIOGRID-SMALL-SCALE-STUDIES |
| RET | GFRA1 | 0.11091611 | Physical Interactions | BIOGRID-SMALL-SCALE-STUDIES |
| DOK6 | RET | 0.39060035 | Physical Interactions | BIOGRID-SMALL-SCALE-STUDIES |
| POU3F2 | SOX10 | 0.3139466 | Physical Interactions | BIOGRID-SMALL-SCALE-STUDIES |
| DOK5 | RET | 0.22503276 | Physical Interactions | BIOGRID-SMALL-SCALE-STUDIES |
| ERBB3 | NRG1 | 0.12902229 | Physical Interactions | BIOGRID-SMALL-SCALE-STUDIES |
| CALCA | ECE1 | 0.32326305 | Physical Interactions | BIOGRID-SMALL-SCALE-STUDIES |
| GGA2 | BACE2 | 0.2304688 | Physical Interactions | BIOGRID-SMALL-SCALE-STUDIES |
| LIMK1 | NRG1 | 0.21574892 | Physical Interactions | BIOGRID-SMALL-SCALE-STUDIES |
| ERBB4 | NRG1 | 0.10640407 | Physical Interactions | BIOGRID-SMALL-SCALE-STUDIES |
| GNA11 | EDNRB | 0.15899238 | Physical Interactions | BIOGRID-SMALL-SCALE-STUDIES |
| DOK4 | RET | 0.08257147 | Physical Interactions | BIOGRID-SMALL-SCALE-STUDIES |
| GGA1 | BACE2 | 0.16921341 | Physical Interactions | BIOGRID-SMALL-SCALE-STUDIES |
| GGA1 | GGA2 | 0.020958507 | Physical Interactions | BIOGRID-SMALL-SCALE-STUDIES |
| PAX3 | SOX10 | 0.11514674 | Physical Interactions | BIOGRID-SMALL-SCALE-STUDIES |
| PAX3 | POU3F2 | 0.121619366 | Physical Interactions | BIOGRID-SMALL-SCALE-STUDIES |
| EDNRA | EDN1 | 0.6836266 | Physical Interactions | BIOGRID-SMALL-SCALE-STUDIES |
| EDNRA | GNA11 | 0.12169884 | Physical Interactions | BIOGRID-SMALL-SCALE-STUDIES |
| NRTN | GFRA2 | 0.7516884 | Physical Interactions | IREF-HPRD |
| GFRA1 | NRTN | 0.4164441 | Physical Interactions | IREF-HPRD |
| EDNRB | EDN3 | 0.20716113 | Physical Interactions | IREF-HPRD |
| RET | NRTN | 0.0996575 | Physical Interactions | IREF-HPRD |
| RET | GFRA1 | 0.12721889 | Physical Interactions | IREF-HPRD |
| DOK6 | RET | 0.3977346 | Physical Interactions | IREF-HPRD |
| POU3F2 | SOX10 | 0.06004467 | Physical Interactions | IREF-HPRD |
| EDN1 | EDNRB | 0.13492642 | Physical Interactions | IREF-HPRD |
| EDN1 | ECE1 | 0.36904925 | Physical Interactions | IREF-HPRD |
| DOK5 | RET | 0.091177195 | Physical Interactions | IREF-HPRD |
| ERBB3 | NRG1 | 0.077375576 | Physical Interactions | IREF-HPRD |
| LIMK1 | NRG1 | 0.10884538 | Physical Interactions | IREF-HPRD |
| ERBB4 | NRG1 | 0.07353566 | Physical Interactions | IREF-HPRD |
| ERBB4 | ERBB3 | 0.0239297 | Physical Interactions | IREF-HPRD |
| GNA11 | EDNRB | 0.10743559 | Physical Interactions | IREF-HPRD |
| DOK4 | RET | 0.10562537 | Physical Interactions | IREF-HPRD |
| NRP2 | SEMA3C | 0.34096777 | Physical Interactions | IREF-HPRD |
| PAX3 | SOX10 | 0.049109634 | Physical Interactions | IREF-HPRD |
| CTSE | EDN3 | 0.2647496 | Physical Interactions | IREF-HPRD |
| KEL | EDN3 | 0.21966153 | Physical Interactions | IREF-HPRD |
| KEL | EDN1 | 0.14306808 | Physical Interactions | IREF-HPRD |
| KEL | EDN2 | 0.3426131 | Physical Interactions | IREF-HPRD |
| EDNRA | EDN3 | 0.23680995 | Physical Interactions | IREF-HPRD |
| EDNRA | EDN1 | 0.15423703 | Physical Interactions | IREF-HPRD |
| EDNRA | GNA11 | 0.122811735 | Physical Interactions | IREF-HPRD |
| GDNF | GFRA2 | 0.53628266 | Physical Interactions | IREF-SMALL-SCALE-STUDIES |
| NRTN | GFRA2 | 0.4510319 | Physical Interactions | IREF-SMALL-SCALE-STUDIES |
| GFRA1 | GDNF | 0.40974185 | Physical Interactions | IREF-SMALL-SCALE-STUDIES |
| GFRA1 | NRTN | 0.34460676 | Physical Interactions | IREF-SMALL-SCALE-STUDIES |
| EDNRB | EDN3 | 0.1896694 | Physical Interactions | IREF-SMALL-SCALE-STUDIES |
| RET | NRTN | 0.0966079 | Physical Interactions | IREF-SMALL-SCALE-STUDIES |
| RET | GFRA1 | 0.06819673 | Physical Interactions | IREF-SMALL-SCALE-STUDIES |
| DOK6 | RET | 0.3477292 | Physical Interactions | IREF-SMALL-SCALE-STUDIES |
| POU3F2 | SOX10 | 0.058273584 | Physical Interactions | IREF-SMALL-SCALE-STUDIES |
| EDN1 | EDNRB | 0.15628514 | Physical Interactions | IREF-SMALL-SCALE-STUDIES |
| EDN1 | ECE1 | 0.23693258 | Physical Interactions | IREF-SMALL-SCALE-STUDIES |
| DOK5 | RET | 0.09001011 | Physical Interactions | IREF-SMALL-SCALE-STUDIES |
| ERBB3 | NRG1 | 0.073802814 | Physical Interactions | IREF-SMALL-SCALE-STUDIES |
| CALCA | ECE1 | 0.17829603 | Physical Interactions | IREF-SMALL-SCALE-STUDIES |
| GGA2 | BACE2 | 0.16888417 | Physical Interactions | IREF-SMALL-SCALE-STUDIES |
| LIMK1 | NRG1 | 0.08647099 | Physical Interactions | IREF-SMALL-SCALE-STUDIES |
| ERBB4 | NRG1 | 0.05406162 | Physical Interactions | IREF-SMALL-SCALE-STUDIES |
| ERBB4 | ERBB3 | 0.016038094 | Physical Interactions | IREF-SMALL-SCALE-STUDIES |
| GNA11 | EDNRB | 0.08702228 | Physical Interactions | IREF-SMALL-SCALE-STUDIES |
| DOK4 | RET | 0.06294114 | Physical Interactions | IREF-SMALL-SCALE-STUDIES |
| NRP2 | SEMA3C | 0.2838962 | Physical Interactions | IREF-SMALL-SCALE-STUDIES |
| GGA1 | BACE2 | 0.1338687 | Physical Interactions | IREF-SMALL-SCALE-STUDIES |
| GGA1 | GGA2 | 0.016479712 | Physical Interactions | IREF-SMALL-SCALE-STUDIES |
| PAX3 | SOX10 | 0.027216258 | Physical Interactions | IREF-SMALL-SCALE-STUDIES |
| PAX3 | POU3F2 | 0.07423616 | Physical Interactions | IREF-SMALL-SCALE-STUDIES |
| CTSE | EDN3 | 0.39691225 | Physical Interactions | IREF-SMALL-SCALE-STUDIES |
| KEL | EDN3 | 0.23978762 | Physical Interactions | IREF-SMALL-SCALE-STUDIES |
| KEL | EDN1 | 0.19758192 | Physical Interactions | IREF-SMALL-SCALE-STUDIES |
| KEL | EDN2 | 0.40380448 | Physical Interactions | IREF-SMALL-SCALE-STUDIES |
| EDNRA | EDN3 | 0.16588858 | Physical Interactions | IREF-SMALL-SCALE-STUDIES |
| EDNRA | EDN1 | 0.13669005 | Physical Interactions | IREF-SMALL-SCALE-STUDIES |
| EDNRA | GNA11 | 0.076111406 | Physical Interactions | IREF-SMALL-SCALE-STUDIES |
| GFRA1 | GDNF | 1 | Predicted | I2D-BioGRID-Rat2Human |
| GDNF | GFRA2 | 0.5305742 | Predicted | Wu-Stein-2010 |
| NRTN | GFRA2 | 0.76536685 | Predicted | Wu-Stein-2010 |
| POU3F2 | SOX10 | 0.07926933 | Predicted | Wu-Stein-2010 |
| LIMK1 | NRG1 | 0.096797444 | Predicted | Wu-Stein-2010 |
| ERBB4 | NRG1 | 0.09195613 | Predicted | Wu-Stein-2010 |
| ERBB4 | ERBB3 | 0.11817376 | Predicted | Wu-Stein-2010 |
| NRP2 | SEMA3C | 0.53790295 | Predicted | Wu-Stein-2010 |
| GGA1 | GGA2 | 0.10785079 | Predicted | Wu-Stein-2010 |
| PAX3 | SOX10 | 0.037514698 | Predicted | Wu-Stein-2010 |
| PAX3 | POU3F2 | 0.0685344 | Predicted | Wu-Stein-2010 |
| NRTN | GDNF | 0.021454364 | Shared protein domains | INTERPRO |
| SEMA3C | SEMA3D | 0.023268186 | Shared protein domains | INTERPRO |
| GFRA1 | GFRA2 | 0.31737545 | Shared protein domains | INTERPRO |
| POU3F2 | PHOX2B | 0.010391838 | Shared protein domains | INTERPRO |
| EDN1 | EDN3 | 0.5 | Shared protein domains | INTERPRO |
| DOK5 | DOK6 | 0.030197645 | Shared protein domains | INTERPRO |
| ERBB3 | RET | 0.008923296 | Shared protein domains | INTERPRO |
| LIMK1 | RET | 0.009555539 | Shared protein domains | INTERPRO |
| EDN2 | EDN3 | 0.5 | Shared protein domains | INTERPRO |
| EDN2 | EDN1 | 0.5 | Shared protein domains | INTERPRO |
| ERBB4 | RET | 0.009827036 | Shared protein domains | INTERPRO |
| ERBB4 | ERBB3 | 0.049965244 | Shared protein domains | INTERPRO |
| DOK4 | DOK6 | 0.030030888 | Shared protein domains | INTERPRO |
| DOK4 | DOK5 | 0.028351294 | Shared protein domains | INTERPRO |
| GGA1 | GGA2 | 0.09857911 | Shared protein domains | INTERPRO |
| PAX3 | PHOX2B | 0.012696284 | Shared protein domains | INTERPRO |
| CTSE | BACE2 | 0.07537251 | Shared protein domains | INTERPRO |
| KEL | ECE1 | 0.18076143 | Shared protein domains | INTERPRO |
| EDNRA | EDNRB | 0.13366154 | Shared protein domains | INTERPRO |
| GFRA4 | GFRA2 | 0.21945418 | Shared protein domains | INTERPRO |
| GFRA4 | GFRA1 | 0.21945418 | Shared protein domains | INTERPRO |
| NRTN | GDNF | 0.031779222 | Shared protein domains | PFAM |
| SEMA3C | SEMA3D | 0.030097127 | Shared protein domains | PFAM |
| GFRA1 | GFRA2 | 0.2 | Shared protein domains | PFAM |
| POU3F2 | PHOX2B | 0.004499285 | Shared protein domains | PFAM |
| EDN1 | EDN3 | 0.5 | Shared protein domains | PFAM |
| DOK5 | DOK6 | 0.110458985 | Shared protein domains | PFAM |
| EDN2 | EDN3 | 0.5 | Shared protein domains | PFAM |
| EDN2 | EDN1 | 0.5 | Shared protein domains | PFAM |
| ERBB4 | ERBB3 | 0.078924544 | Shared protein domains | PFAM |
| DOK4 | DOK6 | 0.058767863 | Shared protein domains | PFAM |
| DOK4 | DOK5 | 0.058767863 | Shared protein domains | PFAM |
| GGA1 | GGA2 | 0.13708399 | Shared protein domains | PFAM |
| PAX3 | PHOX2B | 0.00469992 | Shared protein domains | PFAM |
| CTSE | BACE2 | 0.08385654 | Shared protein domains | PFAM |
| KEL | ECE1 | 0.1735997 | Shared protein domains | PFAM |
| EDNRA | EDNRB | 0.00354377 | Shared protein domains | PFAM |
| GFRA4 | GFRA2 | 0.2 | Shared protein domains | PFAM |
| GFRA4 | GFRA1 | 0.2 | Shared protein domains | PFAM |
